# Dual mechanisms regulate the nucleocytoplasmic localization of human DDX6

**DOI:** 10.1038/srep42853

**Published:** 2017-02-20

**Authors:** Jo-Hsi Huang, Wei-Chi Ku, Yen-Chun Chen, Yi-Ling Chang, Chia-Ying Chu

**Affiliations:** 1 Department of Life Science, College of Life Science, National Taiwan University, Taipei 10617, Taiwan; 2 School of Medicine, College of Medicine, Fu Jen Catholic University, New Taipei 24205, Taiwan; 3 Center for Systems Biology, National Taiwan University, Taipei 10617, Taiwan

## Abstract

DDX6 is a conserved DEAD-box protein (DBP) that plays central roles in cytoplasmic RNA regulation, including processing body (P-body) assembly, mRNA decapping, and translational repression. Beyond its cytoplasmic functions, DDX6 may also have nuclear functions because its orthologues are known to localize to nuclei in several biological contexts. However, it is unclear whether DDX6 is generally present in human cell nuclei, and the molecular mechanism underlying DDX6 subcellular distribution remains elusive. In this study, we showed that DDX6 is commonly present in the nuclei of human-derived cells. Our structural and molecular analyses deviate from the current model that the shuttling of DDX6 is directly mediated by the canonical nuclear localization signal (NLS) and nuclear export signal (NES), which are recognized and transported by Importin-α/β and CRM1, respectively. Instead, we show that DDX6 can be transported by 4E-T in a piggyback manner. Furthermore, we provide evidence for a novel nuclear targeting mechanism in which DDX6 enters the newly formed nuclei by “hitch-hiking” on mitotic chromosomes with its C-terminal domain during M phase progression. Together, our results indicate that the nucleocytoplasmic localization of DDX6 is regulated by these dual mechanisms.

The DDX6 protein family is evolutionarily and functionally conserved among eukaryotes^1^,^2^. DDX6 homologues share a high degree of peptide sequence similarity within the helicase core^1^,^2^, indicating conservation at the structural, interactional, and functional levels. Structurally, DDX6 proteins are composed of two RecA-like domains, which contain helicase motifs that are crucial to the ATPase and RNA-binding activities^1^,^2^. At the interaction level, DDX6 homologues interact with multiple post-transcriptional regulators, including the miRNA-induced silencing complex (miRISC)^3^,^4^,^5^,^6^, the PATL1-LSM_1–7_ complex^7^,^8^,^9^, and the decapping complex^8^,^9^,^10^. Functionally, DDX6 homologues are required for efficient gene silencing downstream of multiple pathways, including miRNA-mediated^3^,^4^,^5^,^6^ and AU-rich element-dependent gene silencing^11^,^12^. Previous research has also demonstrated that DDX6 homologues can facilitate both general and targeted mRNA decay via the decapping pathway^13^,^14^,^15^,^16^,^17^. In the absence of active decapping machinery, DDX6 homologues can still silence protein expression through translational repression^14^. Moreover, DDX6 deregulation can alter translational status in various biological contexts^3^,^18^. At the cellular level, silenced RNA, translational repressors, and decay factors can assemble into P-bodies as a consequence of gene silencing^19^. P-body assembly and maintenance strictly depend on DDX6 even under arsenite-induced stress^20^, reflecting the central role of DDX6 post-transcriptional regulation.

DDX6 has known functions in the cytoplasm, but there is also evidence from various model organisms indicating that DDX6 homologues have functions in the nucleus beyond their role in cytoplasmic mRNA silencing. In the yeast *Saccharomyces cerevisiae*, Dhh1 associates with RNA polymerase II and can affect transcription^21^,^22^. In the planarian *Dugesia japonica*, DjCBC-1 is present in the nuclei of neoblasts^23^. In *Xenopus laevis*, Xp54 is present in the nuclei of early oocytes^24^. In *Rattus norvegicus*, DDX6 is also observed in the nuclei of Leydig cells^25^. In human cells, DDX6 was detected in the nuclear extracts of MKN45 gastric cancer cells by western blot (WB)^26^ and in the nuclei of *Dystrophia myotonica* (DM)-affected fibroblasts by immunofluorescence (IF)^27^. However, it is unclear whether the nuclear presence of DDX6 is restricted to specific cells, namely the MKN45 and DM-affected cells, and the nuclear functions for DDX6 homologues are still undetermined.

Moreover, the mechanism underlying DDX6 subcellular distribution remains elusive. A previous study has proposed that vertebrate DDX6 homologues use a lysine/arginine-rich nuclear localization signal (K/R-rich NLS; referred to as the putative NLS) and a leucine-rich nuclear export signal (L-rich NES; referred to as the putative NES) for nucleocytoplasmic shuttling^1^,^24^. To our knowledge, there is currently no experimental evidence supporting the functionality of the putative NLS. Furthermore, the evidence for the putative NES is unconvincing; there are conflicting data in the current literature. The original study on shuttling behaviour demonstrated that the N-terminal 1–164 segment of Xp54, harbouring both the putative NLS and NES, can shuttle nucleocytoplasmically^24^. However, the same study also reported that the distribution of over-expressed full length (FL) Xp54 is restricted to the cytoplasm and is insensitive to leptomycin B (LMB)^24^, a potent and irreversible inhibitor for the CRM1 protein. Other studies have also shown that DDX6 is insensitive to LMB treatment^28^,^29^,^30^, and one recent study has reported decreased DDX6 levels in cytoplasmic extracts following LMB treatment^26^. Because the subcellular distribution and its underlying mechanisms can affect the functions of cellular protein, these conflicts and discrepancies limit our understanding of nuclear DDX6.

In this study, we examined the nuclear presence of DDX6, assessed its interaction with nuclear lncRNA, and dissected the mechanism controlling the subcellular distribution of DDX6. We show that DDX6 is present in the nuclei of human cell models and interacts with nuclear lncRNA MALAT1. Our subcellular distribution results stand in contrast to the existing nucleocytoplasmic shuttling model. We show that the putative NES is masked by protein folding, resulting in its inaccessibility to CRM1, the mediator protein for the L-rich NES-dependent export. We also provide the first experimental evidence to clarify the validity of the putative NLS. Alternatively, we demonstrate that the DDX6 C-terminal domain (CTD) can facilitate nuclear localization through “hitch-hiking” on mitotic chromosomes. Interestingly, with the overexpression of EIF4E nuclear import factor 1/transporter (EIF4ENIF1/4E-T), a protein-protein interacting partner of DDX6, DDX6 can also be distributed throughout the nucleus and cytoplasm in a shuttling-dependent manner. Collectively, our results support parallel mechanisms for the nucleocytoplasmic distribution of DDX6 in dividing cells, and provide insights into the co-operating mechanisms for the nuclear shuttling of DBPs and other RNA-binding proteins in human cells.

## Results

### Nuclear localization of DDX6 in human cells

**Figure 1:**
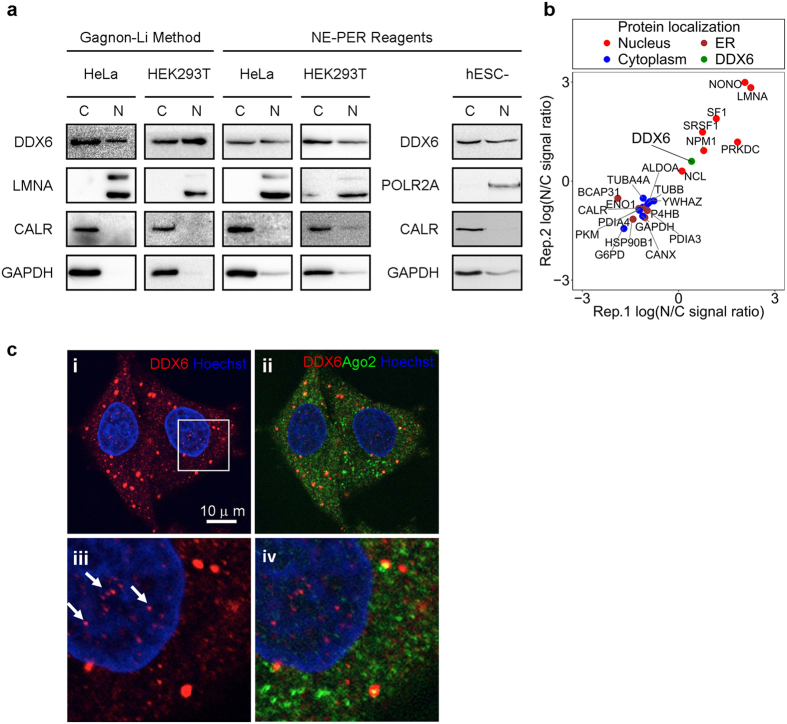
DDX6 is present in human cell nuclei. (**a**) DDX6 is detectable in the nuclear extracts of human cell lines. Cytoplasmic and nuclear extracts from HeLa cells, HEK293T cells, and hESC-derived CM were prepared by NE-PER reagent or Gagnon-Li method. Equal amounts (proteins in μg) of nuclear and cytoplasmic extracts were separated by SDS-PAGE and analyzed by WB. LMNA and POLR2A p-Ser2: lamin A/C and phosphorylated RNA polymerase II subunit 1, nuclear markers; GAPDH: a cytosolic marker; CALR: an ER lumen marker. (**b**) Quantitative MS analysis confirms the nuclear presence of DDX6. Scatter plot showing the comparison of the protein nucleocytoplasmic signal ratios between the two replicates of the quantitative MS data. The original samples were fractionated by NE-PER to yield cytoplasmic and nuclear extracts as in **(a)**. The N/C signal ratios were calculated using the summed-up extracted ion chromatogram (XIC) intensities, an estimate of the protein abundance. Several known cytosolic (blue), ER (brown), and nuclear (red) proteins were shown side-by-side with DDX6 (green). (**c**) IF detects endogenous DDX6 in the nuclei of human cells. HeLa cells were analyzed by immunofluorescence using antibodies against DDX6 (shown in red) and AGO2 (shown in green). Nuclei were stained with Hoechst 33342 (shown in blue) and images were digitally merged **(c**, i and ii). Bottom panels (**c**,iii and iv) display magnified views of the boxed areas in i. Arrows indicated endogenous DDX6 in the nuclei. Scale bar = 10 μm.

To examine the nuclear presence of DDX6 in human cell nuclei, we performed subcellular fractionation followed by WB on the HeLa human cervical cancer cell line, the transformed human embryonic kidney cell line HEK293T, and human embryonic stem cell-derived cardiomyocytes (hESC-derived CM). We used the Gagnon-Li method^31^,^32^ and the NE-PER reagents for nuclear/cytoplasmic fractionation (Fig. 1a). Our WB results showed that DDX6 was detectable in both cytoplasmic and nuclear extracts prepared using both methods (Fig. 1a). The specificity of anti-DDX6 WB in nuclear extract was further confirmed in DDX6-depleted cells (Fig. S1a and b). The WB results also showed specific detection of the nuclear marker LMNA in the nuclear extracts, whereas the cytoplasmic marker GAPDH and endoplasmic reticulum (ER) marker CALR were barely detected (Fig. 1a). Notably, LMNA was expressed at a low level in hESC-derived CM, so we used POLR2A p-Ser2 as a nuclear fraction marker instead (Fig. 1a). These results confirmed that there was minimal cross-contamination between the cytoplasmic and nuclear fractions, supporting the nuclear presence of DDX6. To the same end, we also exploited an in-house HeLa nuclear/cytoplasmic proteome dataset with two biological replicates that was produced by quantitative mass spectrometry (Fig. 1b). In both replicates, DDX6 was found to be present based on at least two unique peptides in both the nuclear and cytoplasmic fractions (Fig. 1b and Table S1). Similar to our WB results, various markers for the nucleus, cytoplasm, or ER were differentially detected in the cytoplasmic and the nuclear fractions (Fig. 1b). This result also supports the nuclear presence of DDX6. In addition, IF staining of DDX6 in HeLa cells followed by confocal microscopy (Fig. 1c) and 3D-projection (Fig. S2) further revealed the nuclear localization of DDX6, and the detected signals of DDX6 in nuclei were less as compared with that in cytoplasm. The specificity of anti-DDX6 IF in the cell nuclei was further confirmed in DDX6-depleted cells and mock IF (Fig. S3). To circumvent the artefacts that could potentially be introduced by fractionation or fixation, we also scanned live HeLa cells expressing YFP-tagged DDX6 (YFP-DDX6 FL) by confocal microscopy and detected fluorescent signals within the nuclear area (Fig. S4). Combined, our results support the nuclear presence of DDX6 in human-derived cells.

To test whether nuclear DDX6 is functionally active for RNA binding, we examined the interaction of DDX6 with nuclear RNAs, in particular the nuclear-retained lncRNAs. To this end, we performed a standard RNA co-immunoprecipitation (RIP) by using anti-DDX6 to pull down the endogenous DDX6 associated complexes and followed by RT-qPCR to examine the DDX6 interacting RNAs. Our results showed that one of the nuclear-retained lncRNAs, MALAT1, was enriched by anti-DDX6 IP (Fig. S5a). The specificity of anti-DDX6 IP was confirmed by control IgG IP (Fig. S5a), anti-DDX6 IP in DDX6-depleted cells, and the co-IP of several known protein-protein interaction (PPI) partners (Fig. S6a). The specificity of MALAT1 co-IP was confirmed in DDX6-depleted cells (Fig. S6b). To further confirm the interaction of DDX6 with MALAT1 *in vivo*, we tracked the co-localization of DDX6 and MALAT1 in interphase HeLa cells with fluorescent *in situ* hybridization (FISH) and IF double staining. DDX6 was frequently observed co-localizing with, or docking to, MALAT1 in cells in which MALAT1 aggregates to nuclear speckles (Figs S5b and S6b,c), supporting the fact that DDX6 interacts with MALAT1 in the nucleus.

### DDX6 is insensitive to LMB treatment

**Figure 2:**
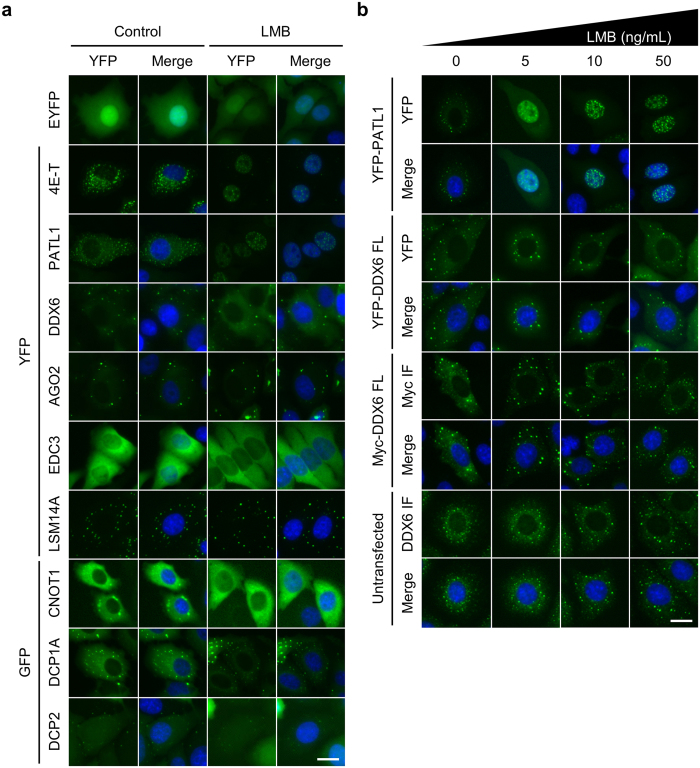
The nucleocytoplasmic localization of DDX6 is insensitive to LMB treatment. (**a**) LMB treatment does not alter DDX6 nucleocytoplasmic distribution. HeLa cells expressing control EYFP, EYFP- or EGFP-tagged P-body components were treated with 5 ng/mL LMB for 5 hours prior to imaging. (**b**) DDX6 is insensitive to high concentration LMB treatment. HeLa cells transfected with YFP-PATL1, YFP-DDX6 FL, and myc-DDX6 FL, and untransfected cells were treated with 0, 5, 10, and 50 ng/mL LMB for 5 hours prior to imaging. (**a** and **b**) Same volume of solvent to highest dose LMB treatment was used as control (0 ng/mL LMB). YFP signal is shown in green. Nuclei were stained with Hoechst (shown in blue). Merge channels show overlay of YFP and Hoechst signals. Scale bar = 20 μm.

Because DDX6 localized to human cell nuclei, we next sought to determine how it is distributed nucleocytoplasmically. Although it was proposed that vertebrate DDX6 homologues are shuttled between the nucleus and the cytoplasm by the importin-α/β and the CRM1 pathways^1^,^24^, this shuttling model was challenged by conflicting data concerning CRM1-dependent export^24^,^28^,^29^,^33^. To clarify the nuclear shuttling mechanism of DDX6, as well as that of other P-body interacting proteins, we surveyed the LMB-sensitive P-body components. Our results showed that only PATL1 and 4E-T, but not DDX6, accumulated in the cell nuclei conspicuously after the treatment of LMB (Fig. 2a). Furthermore, even under a high dose of LMB, no grossly observable nuclear accumulation was seen for DDX6 (Figs 2b and S7). Together, our results indicate that LMB does not affect DDX6 and its nuclear export via the CRM1-dependent pathway.

### The putative NES is structurally masked and is inaccessible to CRM1

**Figure 3:**
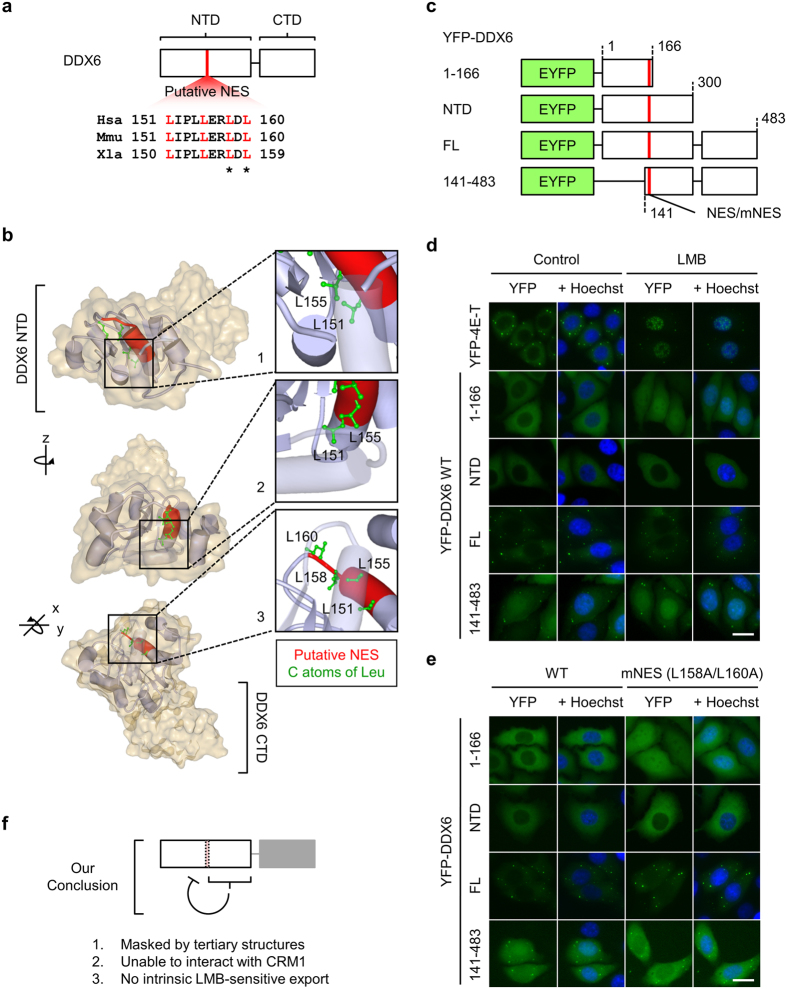
The putative NES is masked by DDX6 NTD folding. (**a**) Illustration of the putative L-rich NES in DDX6. The putative NES spans position 151–160 in DDX6 NTD (red segment). The white box represents DDX6 sequence. Sequence alignment of human, mouse and *Xenopus* DDX6 homologs is shown. Leucine residues matching the consensus L-rich NES pattern is colored in red. Asterisks denote the leucine residues targeted for mutagenesis (L158 and L160; L to A substitution). Hsa: *Homo sapiens*; Mmu: *Mus musculus*; Xla: *Xenopus laevis*. (**b**) The putative NES is buried in the core of stably folded DDX6 NTD. DDX6 in ATPase-incompatible conformation (4CT5) is visualized, colored, rotated to highlight the structural features of the putative NES. Cylinders represent α-helices; flat arrows represent β-sheets. NES peptide backbone is colored in red; carbon atoms of leucine residues are colored in green. Insets display magnified views of the boxed areas near the putative NES. (**c**) Illustration depicts the constructs of YFP-DDX6 1–166, YFP-DDX6 NTD, YFP-DDX6 FL, and YFP-DDX6 141–483. Various segments of DDX6 were N-terminally tagged with EYFP. Region of the putative NES is shown in red. All NES mutants (mNES) bear L158A/L160A substitutions. (**d**) Nucleocytoplasmic distribution of YFP-DDX6 NTD and YFP-DDX6 FL are insensitive to LMB treatment. HeLa cells expressing YFP-4E-T, YFP-DDX6 1–166 WT, YFP-DDX6 NTD WT, YFP-DDX6 FL WT and YFP-DDX6 141–483 WT were treated with 5 ng/mL LMB for 5 hr prior to imaging. Same volume of 70% methanol (MeOH; solvent) was used as control treatment. (**e**) Nucleocytoplasmic distributions of YFP-DDX6 NTD, YFP-DDX6 FL, and YFP-DDX6 141–483 are insensitive to putative NES mutation. YFP-DDX6 1–166, YFP-DDX6 NTD, YFP-DDX6 FL, and YFP-DDX6 141–483 WT and mNES were transiently expressed in HeLa cells. YFP signal is shown in green. Nuclei were stained with Hoechst (shown in blue). Scale bar = 20 μm. (**f**) Diagram shows our conclusion for the accessibility of the putative NES.

To support our new evidence that DDX6 is not LMB-sensitive, we resorted to a structural analysis to obtain biochemical insight into the feasibility of CRM1-dependent export of DDX6. Inspection of the existing X-ray crystallographic structures showed that the DDX6 NTD folds into a globular structure in all three published crystal structures: DDX6 NTD (1VEC)^34^, ATPase-compatible conformation (4CT4)^4^, and the ATPase-incompatible conformation (4CT5)^4^. The putative NES proposed by previous studies (Fig. 3a) indeed folds into an L-rich NES-like structure that is characterized by α-helical folding with leucine residues protruding in the same direction^35^ (Figs 3b and S8). Nonetheless, this putative NES is buried inside the core region of the DDX6 NTD, with L151 and L155 directly blocked by the 174–199 segment (Fig. 3b inset 1 and 2 and Fig. S8), while L158 and L160 protrude downward and inward to the DDX6 NTD core (Fig. 3b inset 3 and Fig. S8). In addition, the putative NES is one of the most stably folded segments within the DDX6 NTD (Fig. S9a and b), indicating that the putative NES is unlikely to contact other proteins at the molecular surfaces.

Despite the structural features, the N-terminal 1–164 segment of Xp54, which is homologous to the 1–166 segment of human DDX6 (Fig. 3a), was reported to be exported by the CRM1-dependent pathway^24^. One plausible explanation for this discrepancy is that the recognition and shuttling of the Xp54 1–164 segment by CRM1 results from the removal of the downstream masking peptide segments, which exposes the putative NES. That is, the CRM1-dependent export of the Xp54 1–164 segment could be a gain-of-function mutation when the protein is truncated. To test this hypothesis, we examined the localization of YFP-DDX6 1–166 and YFP-DDX6 141–483, two truncated DDX6 mutants. We predicted that, whereas the 1–166 segment of human DDX6 can be exported through the CRM1-dependent pathway, the DDX6 FL, DDX6 NTD, and DDX6 141–483 segments cannot be exported through the CRM1-dependent pathway (Fig. 3c). As expected, the export of YFP-DDX6 1–166 was sensitive to LMB treatment and to mutation at the putative NES (Fig. 3d and e). The localizations of YFP-DDX6 FL, CTD, and 141–483, however, were not altered by LMB treatment or mutation (Fig. 3d and e). These results corroborated our prediction and confirmed that the putative NES is structurally inaccessible to CRM1 in the intact DDX6 NTD. Additionally, the comparative analysis of the homologous regions of the putative NES in other DBPs in the eIF4A-like clade revealed that this segment is highly enriched with hydrophobic amino acids (AA) arranged in NES-like sequences (Fig. S9c–e). However, no NES-like function has been reported for these homologous regions. These findings suggest that the N-terminal region containing the putative NES of DDX6 may instead provide essential hydrophobic interactions for the domain folding common to other DBPs.

### The putative K/R-rich NLS, miRISC, and MALAT1 are dispensable for the nuclear entry of DDX6

The shuttling model from a previous study proposed that the vertebrate DDX6 homologues are imported to the cell nuclei by the importin-α/β complex, which recognizes the K/R-rich NLS^1^,^24^. However, this has not yet been supported by experimental data. To test this model, we mutated the putative NLS (mNLS) in YFP-DDX6 1–166 (Fig. S10a). Upon LMB treatment, YFP-DDX6 1–166 mNLS still accumulated in the nucleus (Fig. S10b), indicating that its ability to enter the nucleus was not determined by the putative NLS. We then used the NucPred program^36^ to scan for K/R-rich NLS embedded within the peptide sequence of DDX6, but no potential target was reported (Fig. S10c). Additionally, treatment with importazole (IPZ), an inhibitor of importin-β, did not block the nuclear localization of endogenous DDX6 and YFP-DDX6 1–166 WT (Fig. S11). The putative NLS of DDX6 deviates from the consensus K/R-rich NLS^37^. These lines of evidence argue against the idea that K/R-rich NLS facilitates the nuclear import of DDX6.

It was recently discovered that the core RNAi machinery can be shuttled nucleocytoplasmically by TNRC6A^29^. Thus, we wondered whether DDX6 is co-transported as part of the miRISC. To this end, we first disrupted RISC formation by AGO2 KD (Fig. S12a) and then examined the nucleocytoplasmic distribution of DDX6 (Fig. S7b). The results showed that DDX6 levels in the cytoplasmic and nuclear extracts were not significantly altered by AGO2 depletion (Fig. S12b). We then tested whether the nuclear localization is regulated by MALAT1. However, we did not observe a significant change in DDX6 levels in the cytoplasmic and nuclear extracts when MALAT1 was depleted (Fig. S12c and d). These data suggest that the nuclear entry of DDX6 is not determined by miRISC or MALAT1 lncRNA.

### DDX6 CTD facilitates nuclear localization

**Figure 4:**
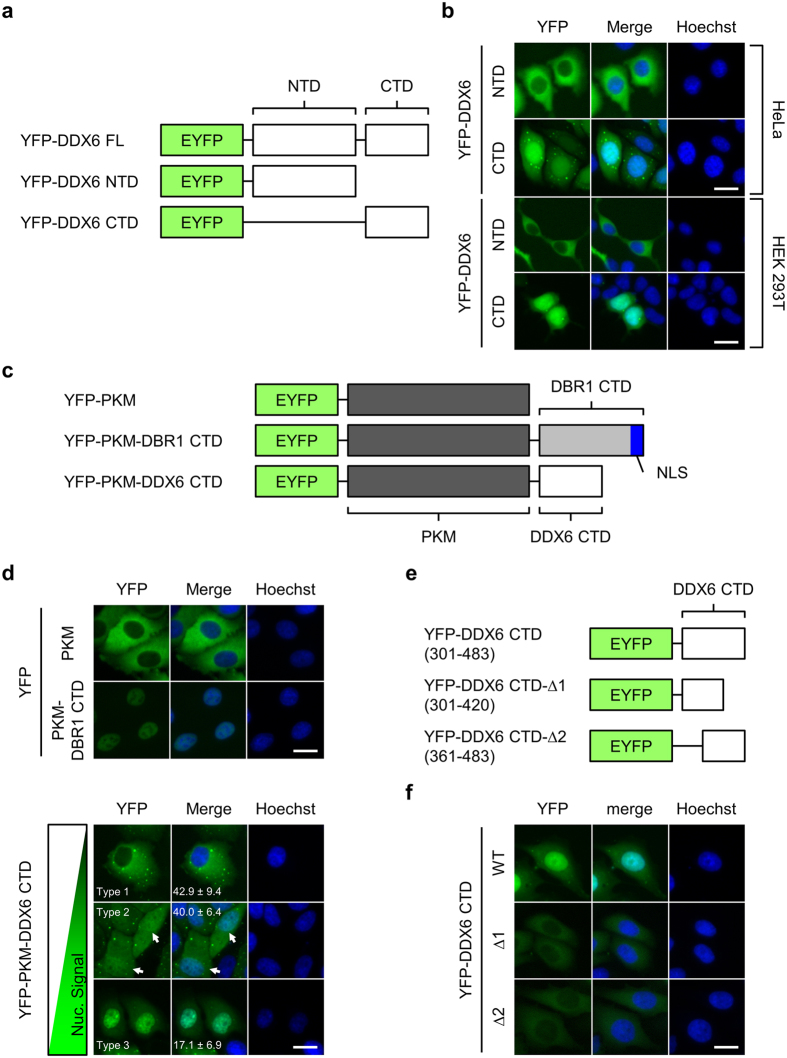
DDX6 CTD determines the nuclear localization of DDX6. (**a**) Illustration depicts YFP-DDX6 FL, NTD, and CTD. NTD: the 1–300 AA; CTD: 301–483 AA. (**b**) YFP-DDX6 CTD localizes to the nuclei of human cells. HeLa and HEK293T cells expressing YFP-DDX6 NTD or YFP-DDX6 CTD were imaged with fluorescent microscope. (**c**) Illustration depicts the YFP-tagged chimeric proteins used for nuclear localization assay. PKM, a non-shuttling cytoplasmic enzyme, was used as the cargo for nuclear transport. ; DBR1 CTD, the CTD of a splicing factor DBR1, harbors a defined K/R-rich NLS, and was used as a positive control. DBR1 CTD and DDX6 CTD were fused to the C-terminus of YFP-PKM. (**d**) YFP-PKM-DDX6 CTD can localize to the nuclei. HeLa cells expressing YFP-PKM, YFP-PKM-DBR1-CTD, or YFP-PKM-DDX6 CTD were imaged 2 days after transfection. A phenotypic continuum of different nucleocytoplasmic distribution exists in the cell population expressing YFP-PKM-DDX6 CTD, and was roughly categorized into 3 types. Type 1: cytoplasm-dominant; type 2: equal cytoplasm/nucleus (indicated by white arrows); type 3: nucleus-dominant. Numbers represent means and standard deviations of the percentage of each type calculated from 3 replicates. **(e**) The diagram showing the truncation mutants of DDX6 CTD. DDX6 CTD was further truncated into CTD-Δ1 (301–420) and CTD-Δ2 (421–483) segments. White box represents DDX6 sequence. (**f**) Deletions in DDX6 CTD abolish the nuclear localization of DDX6. HeLa cells expressing YFP-DDX6 CTD, YFP-DDX6 CTD-Δ1 and YFP-DDX6 CTD-Δ2 were imaged with fluorescent microscope. **(b**,**d** and **f**) YFP signal is shown in green. Nuclei stained with Hoechst is shown in blue. Scale bar = 20 μm.

To map the DDX6 compartment responsible for nuclear entry, we first tracked the subcellular localization of the two DDX6 domains (Figs 4a and S13a). Surprisingly, YFP-DDX6 CTD, similar to YFP-DDX6 FL, was present in the nuclear extract, whereas YFP-DDX6 NTD was barely detectable (Figs 4b and S13b). To rule out the possible effect of nuclear entry driven by the EYFP tag, we verified DDX6 CTD localization with Myc-tagged DDX6 CTD (Myc-DDX6 CTD; Fig. S13a). We found that Myc-DDX6 CTD localized predominantly to the nuclei in a similar pattern to that of YFP-DDX6 CTD (Fig. S13c). Although we noticed that the nucleolar localization of DDX6 CTD could be an artefact resulting from 4% PFA fixation (Fig. S13d), fixation did not change the general nuclear localization of YFP- or Myc-tagged DDX6 CTD. Together, these results suggest that DDX6 CTD can be important for nuclear entry.

To elucidate the role of DDX6 CTD in nuclear localization, we adopted a chimeric protein approach^38^,^39^ to examine whether DDX6 CTD is sufficient to drive nuclear entry. We constructed YFP-PKM-DDX6 CTD and YFP-PKM-DBR1 CTD by fusing DDX6 CTD and DBR1 CTD to the C-terminus of YFP-PKM, a non-shuttling cytoplasmic enzyme (Fig. 4c and d). Unlike YFP-PKM, which predominantly localized to the cytoplasm, YFP-PKM-DBR1 CTD was highly nuclear-enriched (Fig. 4d). These data confirmed that YFP-PKM can be imported by C-terminally fused protein. Interestingly, YFP-PKM-DDX6 CTD exhibited a phenotypic continuum (Fig. 4d). We defined three subcellular localization types: Type 1 represents cells with a dominant cytoplasmic distribution; type 2 represents cells with a homogenous distribution; and type 3 represents cells with a dominant nuclear distribution. Two days after transfection, cells with an apparent nuclear signal (type 2 and 3) accounted for approximately 60% of the transfected cells. Together, these results indicate that DDX6 CTD facilitates nuclear entry.

Because DDX6 CTD provides binding sites for its interacting partners (Fig. S14a), including CNOT1^4^,^5^,^6^, PATL1^7^,^9^,^40^,^41^, 4E-T^40^,^41^, LSM14A^10^,^41^, and EDC3^9^,^10^,^41^, we mutated these sites to test whether nuclear localization depends on an interaction with these proteins. None of the mutants showed strongly abrogated nuclear localization (Fig. S14b). Similarly, mutations in conserved helicase motifs or in consensus phosphorylation sites (Fig. S14a) did not abolish nuclear localization (Fig. S14c and d). Together, our results suggest that these factors are dispensable for DDX6 nuclear localization.

Alternatively, we tested whether DDX6 CTD harbours one or more non-canonical NLSs. These sequences are diverse in terms of length and sequence pattern. We first constructed deletion mutants by further deleting the first 60 and the last 63 amino acids in DDX6 CTD (YFP-DDX6 CTD-Δ1 and YFP-DDX6 CTD-Δ2; Fig. 4e). As a result, the nuclear localizations of both YFP-DDX6 CTD-Δ1 and YFP-DDX6 CTD-Δ2 were abolished (Fig. 4f). The abolition of nuclear localization indicates the following: First, integral DDX6 CTD may be necessary for nuclear localization; second, both the DDX6 301–360 and the 421–483 segments alone were insufficient for nuclear entry, suggesting that these two segments do not function as stand-alone NLSs.

### DDX6 CTD facilitates chromosome association during mitosis

**Figure 5:**
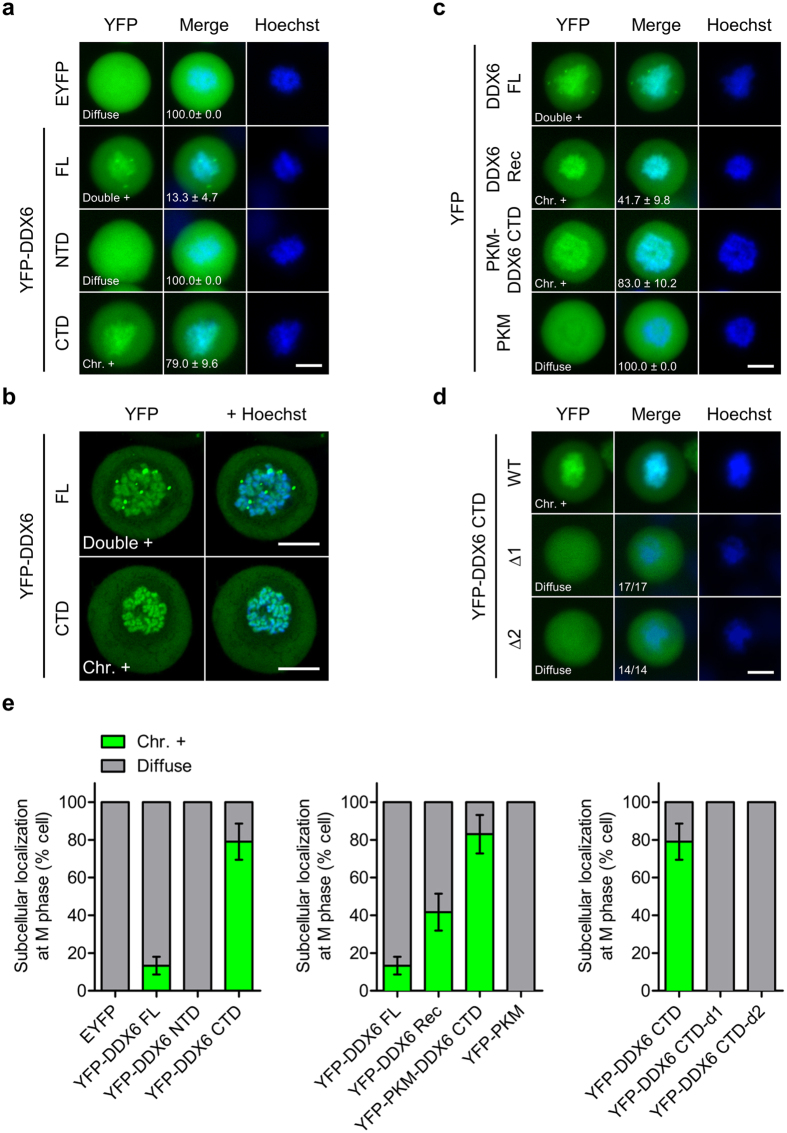
DDX6 FL and CTD bind to the condensed chromosomes during M phase. (**a**) YFP-DDX6 FL and CTD were associated with mitotic chromosomes. HeLa cells transfected with EYFP, YFP-DDX6 FL, -NTD, or -CTD were synchronized at prometaphase using nocodazole (50 μg/mL) and imaged. Images were acquired with fluorescent microscope **(a)** and confocal microscope **(b)**, respectively. **(c)** DDX6 CTD accounts for mitotic chromosome association. Synchronized HeLa cells expressing YFP-DDX6 FL, YFP-DDX6 Rec (Fig. S15), YFP-PKM-DDX6 CTD, and YFP-PKM were imaged with fluorescent microscope. **(d**) Truncation in DDX6 CTD abolishes chromosome association. HeLa cells expressing YFP-DDX6 CTD-Δ1 and -Δ2 were synchronized and imaged with fluorescent microscope. Fractions represent numbers of cells with displayed phenotypes over total counted cells. **(e**) Bar graph showing the phenotype statistics. Data was calculated from three biological replicates as shown in **(a**, **c** and **d**). (**a**–**d**) YFP signal is shown in green. Mitotic chromosomes were stained with Hoechst. Scale bar = 10 μm. Granule+: granule-positive phenotype. Double+: granule/chromosome double-positive phenotype. Chr.+: chromosome-positive phenotype. **(a**, **c** and **d**) Numbers represent means and standard deviations of the percentage of the displayed phenotypes calculated from 3 replicates.

Two other mechanisms could potentially underlie the nuclear localization of DDX6. First, diffuse DDX6 can be engulfed into the nucleus as the nuclear membrane reassembles in late telophase. However, this model is insufficient to explain the predominantly nuclear-localized phenotype of DDX6 CTD (Fig. 4b). We then considered a second possibility, that DDX6 CTD may achieve nuclear entry by “hitch-hiking” on mitotic chromosomes during M phase. Two independent proteomic studies, which detected DDX6 in the mitotic chromosome extract of extract of chicken DT40 cells^42^ and chromatin extracts from human HeLa-S3 cells^43^, provided evidence for this hypothesis. To examine whether DDX6 indeed associates with chromosomes, we tracked the localization of DDX6 constructs in mitotic HeLa cells (Fig. 5a). Whereas EYFP and YFP-DDX6 NTD were diffuse throughout the cytoplasm during M phase, both YFP-DDX6 FL and CTD co-localized with chromosomes (Fig. 5a and f). Viewed by confocal microscopy, YFP-DDX6 FL and CTD displayed an indistinguishable chromosome binding pattern (Fig. 5b). These findings indicated that DDX6 CTD could facilitate binding to mitotic chromosomes.

To confirm that DDX6 CTD is responsible for chromosome association, we used the “domain swap” approach. We first reconstituted YFP-DDX6 (YFP-DDX6 Rec) by fusing the DDX6 CTD to the C-terminus of YFP-DDX6 NTD (Fig. S15a and c). Subsequently, we swapped the DDX6 NTD for PKM. As a result, while YFP-DDX6 Rec was chromosome-positive in 40% of the mitotic cells, YFP-PKM-DDX6 CTD was chromosome-positive in 80% of the mitotic cells (Fig. 5e). This result suggests that the DDX6 NTD is dispensable, or even inhibitory, to chromosome association. Conversely, when the DDX6 CTD was removed, the resulting YFP-PKM was exclusively diffuse in mitotic cells (Fig. 5c and e), suggesting that PKM does not contribute to chromosome binding. We further tested whether the truncation in the DDX6 CTD abolishes the chromosome binding. As a result, both YFP-DDX6 CTD-Δ1 and YFP-DDX6 CTD-Δ2 exhibited a diffuse pattern (Fig. 5d and e), suggesting that structurally integral DDX6 CTD may be required for chromosome association. Collectively, the chromosome binding in the mitotic cells showed a striking positive correlation with the nuclear localization in interphase cells, implying a causal link between these two phenomena.

### DDX6 CTD facilitates nuclear entry by hitchhiking on mitotic chromosomes

**Figure 6:**
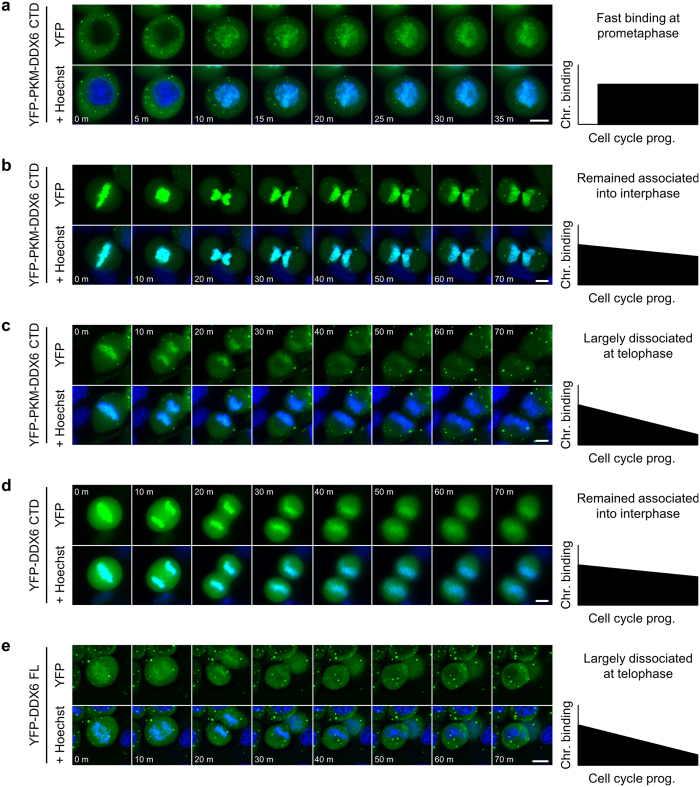
The CTD of DDX6 facilitates nuclear entry via hitchhiking on the mitotic chromosomes. **(a**–**e)** The representative images of the YFP-DDX6 constructs in the time-lapse imaging. The HeLa cells expressing YFP-PKM-DDX6 CTD entering **(a)** or exiting M phase **(b** and **c)** were shown. The HeLa cells expressing YFP-DDX6 CTD **(d)** or YFP-DDX6 FL **(e)** exiting M phase were also shown. The time interval = 5 min in **(a)**; the time interval = 10 min in **(b**–**e)**. The YFP signals were shown in green. The cell nuclei/chromosomes were stained with Hoechst (blue). The diagrams on the right represent the trend in chromosome binding during the cell cycle progression.

To further investigate the relationship between the chromosome binding and nuclear localization of DDX6, we monitored the chromosome-association dynamics of DDX6 constructs during M phase with time-lapse microscopy. First, we observed that cytoplasmic YFP-PKM-DDX6 CTD quickly bound the condensed chromosomes after the nuclear membrane broke down (Fig. 6a). This finding demonstrated that the binding of the mitotic chromosome occurs at early prometaphase, and the subcellular localization of YFP-PKM-DDX6 CTD in the previous cell cycle does not limit this process. We also observed that, in some cells, the interaction between YFP-PKM-DDX6 CTD and the mitotic chromosomes persisted even after cytokinesis (Fig. 6b). Consequently, the chromatin-bound YFP-PKM-DDX6 CTD became trapped in the newly formed nuclei (Fig. 6b). In other cells, the interaction between YFP-PKM-DDX6 CTD and the mitotic chromosomes declined during the mitosis (Fig. 6c), leaving a small amount of YFP-PKM-DDX6 CTD to be enclosed in the newly formed nuclei. This divergent dynamics during the mitosis was consistent with the divergent phenotypic continuum in the interphase cells (Fig. 4d). Conversely, we also observed a persistent interaction between YFP-DDX6 CTD and the mitotic chromosomes, and the subsequent entrapment of YFP-DDX6 CTD in the newly formed nuclei (Fig. 6d). For YFP-DDX6 FL, the interaction with the mitotic chromosomes declined during the mitosis (Fig. 6e). These observations were also consistent with our previous observations in the interphase cells (Figs 2, 3e and 4b). Collectively, our findings point to a novel mechanism in which DDX6 enters newly formed cell nuclei by hitch-hiking on the mitotic chromosomes using its CTD.

### 4E-T can mediate piggyback shuttling of DDX6

**Figure 7:**
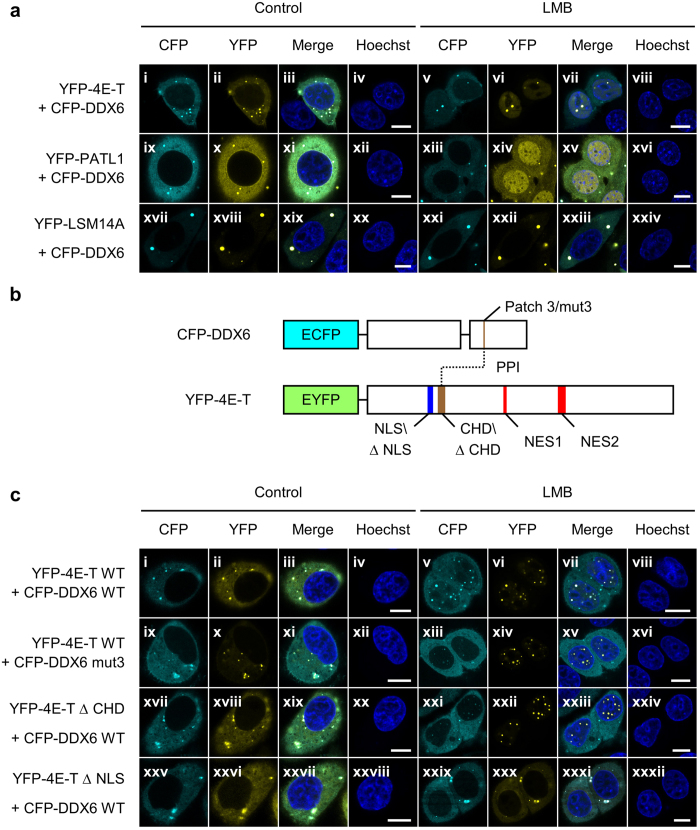
DDX6 can be shuttled by 4E-T in a piggyback manner. (**a**) The representative images of CFP-DDX6 co-expressed with YFP-4E-T, YFP-PATL1, or YFP-LSM14A under the LMB treatment. Overexpression of YFP-4E-T, -PATL1, or -LSM14A elevated the total abundance of these three proteins in HeLa cells. The transfected HeLa cells were treated with LMB (5 ng/mL) or solvent control for 5 hr prior to imaging. Under the LMB treatment, the strong accumulation in the nuclear YFP-4E-T granules **(a**,vi) and the mild elevation of the nuclear CFP-DDX6 level were observed (**a**,v). **(b)** Illustration depicts WT and mutants of CFP-DDX6 and YFP-4E-T. The brown segments represent binding surfaces between DDX6 and 4E-T, namely the patch 3 surface of DDX6 and the CHD of 4E-T. The blue segment represents the validated K/R-rich NLS of 4E-T. And the red segments represent the known L-rich NES in 4E-T. Mut3, the patch 3 mutant, includes three point mutations, namely S343D, Q345D, and R346D. ΔCHD and ΔNLS mutants of 4E-T contain deletions from E217 to D240 and from R195 to N211, respectively. **(c)** The representative images for the co-expressed WT and mutants of CFP-DDX6 and YFP-4E-T. The cells were subjected to LMB treatment prior to imaging as described in **(a)**. The interaction mutants, CFP-DDX6 mut3 and YFP-4E-T ΔCHD, did not affect the localization of YFP-4E-T to nuclear granules **(c**, xiv and xxii), but reduce the localization of CFP-DDX6 to nuclear granules **(c**, xiii and xxi). ΔNLS, the nuclear import mutant, of YFP-4E-T abolished both the nuclear import and nuclear granule localization of both YFP-4E-T and CFP-DDX6 **(c**, xxix and xxx). (**a** and **c**) Nuclei were stained with Hoechst. Scale bar = 20 μm.

It has been recently proposed that DDX6 may be shuttled nucleocytoplasmically in a piggyback style^33^. Among the known interacting partners of DDX6, 4E-T, PATL1, and LSM14B are indeed directly transported by the CRM1 pathway (Fig. 2a)^28^,^33^,^44^. However, several proteomic profiling efforts have shown that DDX6 has a greater average copy number per cell than these other proteins (Fig. S16), especially in HeLa cells (Fig. S16d)^33^,^45^,^46^,^47^. This is consistent with the observation that the DDX6 localization was unaffected under LMB treatment (Fig. 2). To overcome this limitation imposed by the protein stoichiometry, we monitored the localization of the co-expressed CFP-DDX6 with the overexpression of YFP-4E-T, YFP-PATL1, and YFP-LSM14A under LMB treatment. Because LSM14A, unlike its paralogue LSM14B^33^, is not a shuttling protein, we observed no change in the subcellular localization of YFP-LSM14A and CFP-DDX6 under LMB treatment (Fig. 7a, xxi and xxiv). Conversely, YFP-PATL1 accumulated in the nuclei under LMB treatment (Fig. 7a, xiii-xvi). However, YFP-PATL1 displayed a diffuse nuclear pattern in nearly all the observed cells (Fig. 7a, xiv). This was drastically different from the pattern without co-expressing CFP-DDX6, which was characterized by localization to nuclear granules (Fig. 2a). In contrast, YFP-4E-T accumulated and localized to the nuclear granules under LMB treatment regardless of the co-expression of CFP-DDX6 (Fig. 7a, vi). Strikingly, CFP-DDX6 co-localized with YFP-4E-T within the nuclear granules under LMB treatment (Fig. 7a, v-viii). This result indicates that DDX6 can be exported via the CRM1-dependent pathway when complexed with 4E-T in the nucleus. Our finding also suggests that there is 4E-T-mediated DDX6 import for two reasons: First, the localization of CFP-DDX6 to nuclear granules under LMB treatment is only observed when YFP-4E-T is co-expressed. Second, there was no grossly observable nuclear accumulation of CFP-DDX6 with a diffuse pattern in other cases, thereby reducing the possibility that CFP-DDX6 was imported by other potential factors but was not recruited to the nuclear granules by 4E-T.

To further assess the role of 4E-T in the nuclear import of DDX6, we examined whether the binding between DDX6 and 4E-T, and more importantly the nuclear import of 4E-T, are crucial for the import of DDX6. To this end, we constructed a 4E-T-binding mutant of DDX6 (CFP-DDX6 mut3)^40^,^41^, a DDX6-binding mutant of 4E-T (YFP-4E-T ΔCHD)^40^,^48^, and an NLS-mutant of 4E-T (YFP-4E-T ΔNLS; Fig. 7b)^44^. We then examined the localization of CFP-DDX6, with the co-expression of various YFP-4E-T mutants, to the nuclear granules under LMB treatment. Consequently, the granular localization of CFP-DDX6 in the nuclei under LMB-treatment was indeed reduced with the mut3 mutation in CFP-DDX6 (Fig. 7c, xiii-xvi) and with the co-expression of YFP-4E-T ΔCHD (Fig. 7c, xxi-xxiv) while the localization of YFP-4E-T to the nuclear granules appeared unaffected (Fig. 7c, xiv and xxii). Furthermore, the ΔNLS mutation in YFP-4E-T abolished its nuclear entry (Fig. 7c, xxx), and the localization of CFP-DDX6 to the nuclear granules was abolished in correlation (Fig. 7c, xxix). These results confirmed that the nuclear import of 4E-T and its binding to DDX6 are both required for the localization of DDX6 to nuclear granules when 4E-T is overexpressed. This supports a model in which DDX6 can be co-transported with 4E-T in a piggyback manner.

## Discussion

In this study, we used different approaches to show that human DDX6 is present in the nucleus. We used WB and MS to detect DDX6 in nuclear extracts from HeLa cells, HEK293T cells, and hESC-derived CM (Fig. 1a and b). We also used confocal microscopy for anti-DDX6 IF (Fig. 1c) and YFP-DDX6 FL live-cell imaging (Fig. S4) to detect nuclear DDX6 in HeLa cells. Our results were consistent with previous reports that DDX6 is present in the nuclei of MKN45 gastric cancer cells and DM1-affected fibroblasts^26^,^27^. Taken together, the nuclear localization of DDX6 has now been verified in five distinct, human-derived cell types. Because DDX6 is ubiquitously expressed in human tissues/cells, we expect the nuclear localization of DDX6 to be widespread in different human cell types. This notion is supported by the detection of DDX6 in the nuclear extracts of hESC-derived CM (Fig. 1b). This notion is also supported by the knowledge that DDX6 family members are widely observable in the nuclei of metazoans^23^,^24^,^25^,^49^, including lab rodents. The fact that DDX6 family members are present in animal cell nuclei across different phyla implies that DDX6 may have conserved or specialized nuclear functions, which would require further investigation.

We can estimate the ratio of endogenous nuclear DDX6 in various cell types. In our WB and MS analysis, we often observed comparable cytoplasmic and nuclear DDX6 signals (Fig. 1a and b). Because the protein abundance in the cytoplasmic and nuclear extracts was approximately 9:1 to 4:1, as prepared by using the NE-PER reagents, we estimated that the molar ratio of cytoplasmic and nuclear DDX6 in HeLa cells does not exceed 4:1. That is, the nuclear DDX6 is unlikely to account for more than 20% of the cellular DDX6. Although this estimate is conservative, it is comparable to the previous estimates. For instance, in stage III *Xenopus* oocytes, nuclear Xp54 accounts for approximately 16.7% of total Xp54^24^. In the rat testis, nuclear DDX6 accounts for ~7% of total DDX6^25^. This estimate was also consistent when we compared the anti-DDX6 WB signal in the cytoplasmic and nuclear extracts prepared from the same number of cells (Fig. S1c). Although these results are largely comparable, the exact proportion of nuclear DDX6 may be organism-, cell type-, or developmental stage-dependent and may vary depending on the means of detection. Collectively, these results indicate that a relatively minor but substantial proportion of cellular DDX6 is consistently present in the nucleus in many, if not all, cell types.

To further characterize nuclear DDX6, we demonstrated that DDX6 can bind nuclear lncRNA (Figs S5 and S6). This result aligns with previous reports that DDX6 can interact with noncoding regions of mRNAs or lncRNAs both *in vitro* and *in vivo*^9^,^26^,^27^,^34^,^50^,^51^,^52^,^53^,^54^,^55^,^56^. This finding is also among the earliest reports of the interaction between DDX6 and the lncRNA that is highly enriched in the nucleus. We note that, while this manuscript was under revision, a study reported the interaction between DDX6 and 7SK nuclear RNA^57^. Because DDX6 generally binds RNA promiscuously^50^,^52^,^53^ and nuclear lncRNA represents a substantial proportion of the human transcriptome^58^,^59^, we propose that DDX6 may bind to various other nuclear lncRNAs. We also speculate that DDX6 may possess regulatory functions for nuclear lncRNAs in addition to cytoplasmic mRNA silencing. Although we observed no apparent functional regulation of MALAT1 stability or subnuclear localization by DDX6 (data not shown), we cannot fully dismiss the possibility that DDX6 may participate in the structural regulation of lncRNA without further investigations, and we will pursue such investigations in the future.

**Figure 8:**
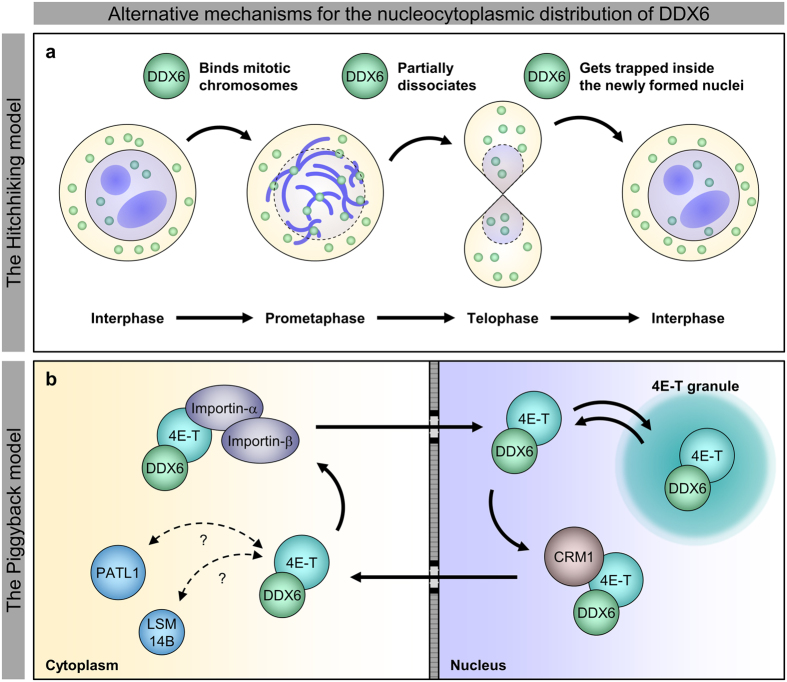
A new synthesis for the nucleocytoplasmic distribution mechanism of DDX6. The diagram summarized the two proposed mechanisms governing the nucleocytoplasmic distribution mechanism of DDX6 in this study. **(a)** The “hitchhiking” model refers to that DDX6 can enter cell nuclei as a passenger on the mitotic chromosomes. **(b)** The “piggyback” model refers to that 4E-T is sufficient to transport DDX6 via the importin-α/β and the CRM1 pathways in a piggyback manner.

We provided evidence contradicting the existing nucleocytoplasmic shuttling model of DDX6, and we revealed a novel nuclear targeting mechanism for DDX6. On one hand, our results, supported by the structural details, show that DDX6 itself is insensitive to LMB treatment; it cannot be exported via the CRM1 pathway with the putative NES (Figs 2 and 3, S7 and S8). On the other hand, our results argue against the role of the K/R-rich NLS in DDX6 nuclear import (Figs S10 and S11). This finding and our structural masking hypothesis for the putative NES together represent a departure from the existing model. Alternatively, our results show that the nuclear import of DDX6 is facilitated by its CTD (Figs 4 and S13), and demonstrate that the DDX6 CTD can mediate association with mitotic chromosomes (Fig. 5). As direct evidence, our time-lapse imaging revealed that the DDX6 CTD indeed enters the nucleus along with post-mitotic chromosomes as the nuclear membrane reassembles (Fig. 6). These findings suggest that DDX6 may enter the cell nucleus by associating with mitotic chromosomes throughout M-phase progression, and we summarized this as the “hitch-hiking” model (Fig. 8a). Under this framework, the association and dissociation processes serve as a mechanism to balance and redistribute DDX6 throughout the cells during mitosis. Furthermore, the incomplete dissociation of DDX6 from mitotic chromosomes underlies the nuclear distribution of DDX6 observed in interphase cells (Fig. 8a). The underlying mechanism and functional relevance of the association with mitotic chromosomes/interphase chromatin and interactions with nuclear lncRNA (Fig. S5 and unpublished data) represent new directions for inquiries into DDX6 functions beyond cytoplasmic mRNA silencing.

In parallel, we also demonstrated that DDX6 can be transported in a piggyback manner through its direct interacting protein, 4E-T (Figs 7 and 8b). This finding enlisted DDX6 as another transport cargo of 4E-T in addition to EIF4E^44^, although we note that the depletion of 4E-T does not greatly alter the nucleocytoplasmic distributions of EIF4E and DDX6 in HeLa cells (Fig. S17). On the other hand, even though we had no explicit evidence for the piggyback style transportation by PATL1, we observed that co-expressed CFP-DDX6 affected the subcellular localization of YFP-PATL1 (Figs 2a and 7a, xiv). Thus, we cannot fully dismiss the possibility that PATL1 is also capable of shuttling DDX6. Moreover, it was originally proposed that *Xenopus* LSM14B (xRAP55B, xRAPB) may serve as a transporter of Xp54^33^. Interestingly, in developing *Xenopus* oocytes, the nuclear Xp54 level decreases after stage III^24^, coinciding with the decline in total xRAPB after stage III^60^. Therefore, it is possible for LSM14B to shuttle DDX6 in human cells, as it is possible for xRAPB to shuttle Xp54 in *Xenopus* oocytes. However, in the biological context of HeLa cells, the abundance of DDX6 greatly exceeds that of PATL1, 4E-T, and LSM14B (Fig. S16). Thus, the shuttling rate by this mechanism may be slow. It is consistent with our observation that the nucleocytoplasmic localization of DDX6 is insensitive to LMB treatment (Fig. 2) and 4E-T depletion in HeLa cells (Fig. S17). Similarly, when we increased the 4E-T levels by overexpressing YFP-4E-T, we observed co-transportation of YFP-4E-T and CFP-DDX6 (Fig. 7). The sensitivity of such co-transportation behaviour to both LMB and the mutation of 4E-T NLS supports the use of CRM1-dependent and importin-α/β-dependent shuttling pathways. Together, these results suggest that this mode of transportation is regulated by protein stoichiometry. Notably, in the oocytes and unfertilized eggs of *Xenopus* (Fig. S16a–c), as well as approximately 25% of the studied human cell lines (Fig. S16e), the protein stoichiometry of DDX6 and 4E-T decreases by one order of magnitude, suggesting the 4E-T-dependent transportation of DDX6 can be more pronounced in these systems.

The two mechanisms of transport were discovered along different lines. The hitch-hiking mechanism stemmed from the rejection of the shuttling model, and the piggyback mechanism was rooted in evidence that supported CRM1-dependent export. Their parallel existence therefore reconciles the conflicting ideas found in past research. Physiologically, the hitch-hiking mechanism is restricted to proliferating cells, whereas the piggyback mechanism is constrained by protein stoichiometry. Our data provide clear evidence that both mechanisms can serve to distribute DDX6 across the nucleus and the cytoplasm. Thus, they may dominate under different biological contexts, and they may complement/buffer each other’s effects.

## Methods

### Cell culture and transfection

HeLa cells and HEK293T cells were cultured in DMEM (GE healthcare) supplied with 10% (v/v) fetal bovine serum, penicillin, streptomycin and 5% CO2 at 37 °C. The conditions for maintaining hESC culture and stimulating CM differentiation were described previously^61^. Lipofectamine 2000 reagent or RNAiMAX reagent (Life Technologies) were used for transfection experiments. To knockdown endogenous DDX6, 4E-T, or AGO2, cells were transfected with 25 nM siRNAs and were harvested at 48 h post-transfection. To knockdown MALAT1, cells were transfected with 50 nM siRNA using RNAiMAX reagent and were harvested at 48 h post-transfection. Cells overexpressing EYFP-tagged or myc-tagged constructs were directly imaged or fixed for IF staining at 24 h post-transfection. For gene expression analyses, cells were harvested at 48 h post-transfection. For siRNA sequences used in this study, see Supplementary Table S2.

### LMB treatment and cell cycle synchronization

Stock LMB (Sigma-Aldrich) solution was prepared at 5 ng/μL concentration in 70% (v/v) methanol (MeOH). Stock IPZ (Sigma-Aldrich) solution was prepared at 10 mM concentration in 100% DMSO. For LMB and IPZ treatment, stock solution was directly diluted in condition media to the desired concentration. Same volume of 70% MeOH or 100% DMSO to the highest dose of LMB or IPZ treatment was used as the control. Cells treated with LMB or IPZ were harvested or imaged at 5 hours post-treatment. For cell cycle synchronization at prometaphase, we adopted the thymidine-nocodazole block strategy. To arrest the cell cycle at S phase, the cells were first incubated with high concentration (2 mM) of thymidine (Sigma-Aldrich) for 24 hours. Next, the cells were released from thymidine treatment for 2 hours prior to nocodazole treatment. Cells were then incubated with 50 ng/mL nocodazole (Sigma-Aldrich) for up to 16 hours to arrest the cell cycle at prometaphase.

### Cloning and constructs

Cloning of full length DDX6, 4E-T, and AGO2 into pEYFP-C1 expression vector was described previously^3^. Deletion mutants and point mutants of DDX6 and 4E-T were generated by either PCR amplification of DNA fragments or 2-step PCR mutagenesis. Myc-tagged DDX6 CTD are PCR amplified from EYFP-tagged constructs and inserted into pKR43 expression vector containing 1 × N-terminal myc tag. Full length PKM, PATL1, EDC3 and DBR1 CTD were PCR-amplified from HeLa cell cDNA, and were subsequently inserted into pEYFP-C1 expression vector. Construction of chimeric proteins was done by inserting DNA fragments into pEYFP-C1 vectors. pEGFP-DCP1A and DCP2 were gifts from Dr. Elisa Izaurralde (Addgene plasmid #25030 and 25031).

### Immunofluorescence (IF) and fluorescent *in situ* hybridization (FISH)

Indirect IF was performed as previously described^3^. In brief, HeLa cells were fixed with 4% (v/v) paraformaldehyde (PFA) in PBS at room temperature for 10 min before penetration with 0.25% (v/v) Triton X-100 in PBS at room temperature for 5 min. The cells were subsequently blocked with 2% (w/v) BSA/0.1% (v/v) Triton X-100 in PBS and were incubated with primary antibody recognizing DDX6 (A300–460A, Bethyl Labratories, Inc), AGO2 (Wako), or with anti-Myc tag (clone 4A6, EMD Millipore) and Alexa Fluor 488 conjugated secondary antibodies (A-21202 or A-21206, Life Technologies).

Custom Stellaris FISH Probes recognizing MALAT1 (NR_002819) were directly labeled with Quasar 570 and were purchased from Biosearch Technologies, Inc. (Petaluma, CA). Probe set sequences utilized in the experiments have been previously described. HeLa cells were hybridized with the MALAT1 Stellaris FISH Probe set, following the manufacturer’s instructions. For anti-DDX6 and anti-phosphorylated SC35 IF (ab11826, Abcam) after MALAT1 FISH, the cells were washed with 50% FISH wash buffer in PBS, PBS and 0.1% Triton X-100 in PBS sequentially before proceeding on with IF procedures from blocking with 2% BSA/0.1% Triton X-100 in PBS.

### Microscopy, image processing and analyses

For general purpose imaging, we routinely use Leica DMI3000 B wide field fluorescent microscope. Images acquired with Leica DMI3000 B were pseudo-colored. Leica DMI3000 B accounts for the images in this article unless specified. Statistics of phenotypic counting were done with ImageJ cell counter plugin. For each construct, at least 3 biological replicates were counted.

To capture high-resolution images of AGO2, nuclear DDX6, MALAT1 and SC35, cells were imaged with Leica confocal imaging system (TCS-SP2) or Zeiss LSM780 confocal microscope. Chromosome association of YFP-DDX6 FL, YFP-DDX6 CTD was also captured with Zeiss confocal microscope. Images were pseudo-colored. For Z-stacking, stacks of images were taken with spacing less than 1 μm along the Z-axis, and were maximally projected on the XY-plane (Z-projection) or projected 3-dimensionally (3D-projection). Z-projection was done using ImageJ Z-projection function. 3D-projection was done with the mesh 3D function in ICY image analysis platform. Signal measurement was carried out using ImageJ. For LUT representation of the optical signals, the built-in Fire LUT in ImageJ was utilized.

For time-lapse imaging, we used DeltaVision Core (GE healthcare) imaging systems. The cells were kept warm (34 °C~37 °C) in heating chamber supplied with CO_2_. The cells were imaged across a 10~20 μm range along the Z axis with 1 μm spacing at 5 minute time interval for at least 3 hours. We utilize the DeltaVision built-in deconvolution function to improve the image resolution post-acquisition. High-resolution images were obtained from applying enhanced ratio (aggressive) mode of blind deconvolution with 10 iterations on each image in the original stacks. YFP, Hoechst and merged signals were made into Montage. The resulting image hyperstacks were Z-projected (sum slice projection) for each time point. The resulting T-stack images were cropped, adjusted, and made into Montage.

### Whole cell, nuclear and cytoplasmic protein extraction

HeLa whole cell lysate, or total cell lysate (TCE), were prepared from lysing cells in RIPA buffer (0.1% [w/v] SDS, 50 mM Tris-HCl pH 7.4, 150 mM NaCl, 1% Nonidet P-40, 1% Triton X-100). Cytoplasmic and nuclear fractions were prepared from HeLa and HEK293T cells using either NE-PER Nuclear and Cytoplasmic Extraction Reagents (Thermo Scientific) or the Gagnon-Li method^31^,^32^. For NE-PER fractionation, the extraction procedure generally follows manufacturer’s protocol, except for the additional washing of nuclei pellet with PBS before resuspending the nuclei with NER reagent. For nuclear lysis, we had two rounds of additional freeze-thawing with liquid nitrogen after four rounds of vortexing/standing described in the original protocol. For Gagnon-Li fractionation, we followed the procedure for preparing cytoplasmic extract and total nuclear extract. In brief, the cytoplasmic fraction was released by lysing cells in hypotonic lysis buffer (HLB; 10 mM Tris-HCl pH 7.5, 10 mM NaCl, 3 mM MgCl_2_, 0.3% [v/v] Nonidet P-40, and 10% [v/v] glycerol). The resulting nuclei pellet was washed with HLB before resuspending the nuclei in nuclear lysis buffer (NLB; 20 mM Tris-HCl pH 7.5, 150 mM KCl, 3 mM MgCl_2_, 0.3% [v/v] Nonidet P-40, and 10% [v/v] glycerol). The nuclei were then lysed by sonication (Digital Sonifier 450, Branson) at 40% power for a total of 45 s with 1 min cooling every 15 s. The nuclear extract was then centrifuged at 1,200 × g for 5 min. The resulting supernatant was collected as total nuclear extract.

### RNA co-immunoprecipitation (RIP)

RIP was performed as described as the previous publication^3^. TCE from HeLa cells was prepared by lysing cells in IP lysis buffer (0.2 mM EDTA, 20 mM HEPES pH 7.9, 0.35% Triton X-100, 10 mM NaCl, 1 mM MgCl_2_) for 10 min on ice and then centrifuged at 12,000 × g for 10 min to remove cell debris. For anti-DDX6 or control IgG IP, the supernatants were subsequently incubated with Dynabeads M-280 Sheep Anti-Rabbit IgG (Life Technologies) and anti-DDX6 (A300–460A, Bethyl Labratories, Inc) or normal rabbit IgG (Santa Cruz) at 4 °C for 3 hr, respectively. After incubation, the beads were washed with IP lysis buffer and IP wash buffer (25 mM Tris-HCl pH 8.0, 150 mM NaCl, 0.3% [v/v] Nonidet P-40) and were split into 2 fractions: one for protein elution and the other for RNA extraction. For protein elution, beads were boiled at 95 °C in SDS buffer. As for RNA extraction, beads were resuspended in TRIzol reagent.

### Western blot

Whole cell lysate, subcellular fractions, and IP eluents prepared as described above were separated by SDS-PAGE and transferred to PVDF membranes (Bio-Rad). Primary antibodies recognizing GFP-tag (Living Colors, Clontech), myc-tag (clone 4A6, EMD Millipore), DDX6 (A300–460A, Bethyl Labratories, Inc), LMNA (ab108595, Abcam), GAPDH (GTX100118, GeneTex), CALR (GTX111627, GeneTex; or, 06–661, EMD Millipore), AGO2 (H00027161-M01, Abnova), 4E-T (A300–706A, Bethyl Laborator), EIF4E (#9742, Cell Signaling), POLR2A p-Ser2 (A300–654A, Bethyl Laboratory), CRM1 (sc-5595, Santa Cruz) and HRP-conjugated secondary antibodies (SC-2004 or SC-2005, Santa Cruz) were used to detect the proteins of interest. For Western blot following IP experiments, HRP-conjugated TrueBlot (18-8816-31, Rockland) anti-rabbit IgG secondary antibody was utilized to avoid detecting denatured IgG heavy chain near 50 KDa.

### RNA extraction, reverse transcription and quantitative real-time PCR (qPCR)

Total RNA from HeLa cells and co-IPed RNA in anti-DDX6 IP experiments were extracted using TRIzol reagent (Life Technologies) and were reverse-transcribed into cDNA using SuperScript III First-Strand Synthesis System (Life Technologies) with random hexamer priming, following the suppliers’ protocols. For RNA extraction from RIP samples, glycogen (molecular biology grade, Roche) was used to promote RNA precipitation according to the supplier’s recommendation. The resulting cDNA were further analyzed with quantitative real-time PCR using iQ SYBR Green Supermix (Bio-Rad) and gene-specific primer sets. Fold changes in gene expression level analyses were calculated by ΔΔC(t) method, using GAPDH mRNA as reference for normalization. Fold enrichments in RIP-qPCR were calculated by 2^[C_IgG_(t) − C_anti-DDX6_(t)]. Primers for qPCR are listed in Supplementary Table S3.

### Quantitative mass spectrometry and analysis

HeLa cells transfected with control siRNA (siDDX6 mm) or DDX6 targeting siRNA (siDDX6 pm). At 48 hours post-transfection, the transfected HeLa cells were harvested and fractionated with NE-PER cytoplasmic and nuclear extraction kit (Thermo Scientific) as described above. Both the nuclear and the cytoplasmic fractions were reduced with dithiothreitol (DTT) and S-alkylated with iodoacetic acid (IAA). Subsequently, the protein samples were digested with endoproteinase Lys-C and trypsin, followed by differential isotopic labeling^62^. The labeled samples were then fractionated with strong cation exchange (SCX) chromatography^63^. The SCX-fractionated samples were subsequently analyzed with liquid chromatography coupled to tandem mass spectrometry. Protein identification was performed with Swiss-Port or RefSeq protein database. Ambiguous peptides were aggregated with Perseus software (http://www.perseus-framework.org/). Protein quantification was performed with MaxQuant software^64^. The spectral intensities were used as estimation of protein abundance for each protein groups, and were log10-transformed and (geometric) mean-centered to give comparable intensity distributions.

### Protein structures, visualization, and K/R-rich NLS prediction

Published crystal structures of DDX6, including apo state DDX6 (4CT5), co-crystalized DDX6 (4CT4, with CNOT1 MIF4G domain), and DDX6 NTD (1VEC), are available at Protein Data Bank (PDB) of Research Collaboratory of Structural Bioinformatics (RCSB). The three crystal structures were downloaded from PDB as pdb files and were visualized with Protein Workshop, an RCSB/PDB protein structure visualization software. The prediction of canonical K/R-rich NLS for DDX6 was performed with the NucPred program implemented as a web-based tool^36^.

## Additional Information

**How to cite this article:** Huang, J.-H. *et al*. Dual mechanisms regulate the nucleocytoplasmic localization of human DDX6. *Sci. Rep.* **7**, 42853; doi: 10.1038/srep42853 (2017).

**Publisher's note:** Springer Nature remains neutral with regard to jurisdictional claims in published maps and institutional affiliations.

## Supplementary Material

Supplementary Information

## References

[b1] Weston A. & Sommerville J. Xp54 and related (DDX6-like) RNA helicases: roles in messenger RNP assembly, translation regulation and RNA degradation. Nucleic Acids Res 34, 3082–3094, doi: 10.1093/nar/gkl409 (2006). 16769775 PMC1477856

[b2] Presnyak V. & Coller J. The DHH1/RCKp54 family of helicases: an ancient family of proteins that promote translational silencing. Biochim Biophys Acta 1829, 817–823, doi: 10.1016/j.bbagrm.2013.03.006 (2013). 23528737 PMC3661697

[b3] Chu C. Y. & Rana T. M. Translation repression in human cells by microRNA-induced gene silencing requires RCK/p54. PLoS Biol 4, e210, doi: 10.1371/journal.pbio.0040210 (2006). 16756390 PMC1475773

[b4] Mathys H. . Structural and biochemical insights to the role of the CCR4-NOT complex and DDX6 ATPase in microRNA repression. Mol Cell 54, 751–765, doi: 10.1016/j.molcel.2014.03.036 (2014). 24768538

[b5] Chen Y. . A DDX6-CNOT1 complex and W-binding pockets in CNOT9 reveal direct links between miRNA target recognition and silencing. Mol Cell 54, 737–750, doi: 10.1016/j.molcel.2014.03.034 (2014). 24768540

[b6] Rouya C. . Human DDX6 effects miRNA-mediated gene silencing via direct binding to CNOT1. RNA 20, 1398–1409, doi: 10.1261/rna.045302.114 (2014). 25035296 PMC4138323

[b7] Ozgur S. & Stoecklin G. Role of Rck-Pat1b binding in assembly of processing-bodies. RNA Biol 10, 528–539, doi: 10.4161/rna.24086 (2013). 23535175 PMC3710359

[b8] Jonas S. & Izaurralde E. The role of disordered protein regions in the assembly of decapping complexes and RNP granules. Genes Dev 27, 2628–2641, doi: 10.1101/gad.227843.113 (2013). 24352420 PMC3877753

[b9] Sharif H. . Structural analysis of the yeast Dhh1-Pat1 complex reveals how Dhh1 engages Pat1, Edc3 and RNA in mutually exclusive interactions. Nucleic Acids Res 41, 8377–8390, doi: 10.1093/nar/gkt600 (2013). 23851565 PMC3783180

[b10] Tritschler F. . Structural basis for the mutually exclusive anchoring of P body components EDC3 and Tral to the DEAD box protein DDX6/Me31B. Mol Cell 33, 661–668, doi: 10.1016/j.molcel.2009.02.014 (2009). 19285948

[b11] Fenger-Gron M., Fillman C., Norrild B. & Lykke-Andersen J. Multiple processing body factors and the ARE binding protein TTP activate mRNA decapping. Mol Cell 20, 905–915, doi: 10.1016/j.molcel.2005.10.031 (2005). 16364915

[b12] Qi M. Y. . AU-rich-element-dependent translation repression requires the cooperation of tristetraprolin and RCK/P54. Mol Cell Biol 32, 913–928, doi: 10.1128/MCB.05340-11 (2012). 22203041 PMC3295194

[b13] Fischer N. & Weis K. The DEAD box protein Dhh1 stimulates the decapping enzyme Dcp1. EMBO J 21, 2788–2797, doi: 10.1093/emboj/21.11.2788 (2002). 12032091 PMC126031

[b14] Carroll J. S., Munchel S. E. & Weis K. The DExD/H box ATPase Dhh1 functions in translational repression, mRNA decay, and processing body dynamics. J Cell Biol 194, 527–537, doi: 10.1083/jcb.201007151 (2011). 21844211 PMC3160580

[b15] Sweet T., Kovalak C. & Coller J. The DEAD-box protein Dhh1 promotes decapping by slowing ribosome movement. PLoS Biol 10, e1001342, doi: 10.1371/journal.pbio.1001342 (2012). 22719226 PMC3373615

[b16] Eulalio A. . Target-specific requirements for enhancers of decapping in miRNA-mediated gene silencing. Genes Dev 21, 2558–2570, doi: 10.1101/gad.443107 (2007). 17901217 PMC2000321

[b17] Nishihara T., Zekri L., Braun J. E. & Izaurralde E. miRISC recruits decapping factors to miRNA targets to enhance their degradation. Nucleic Acids Res 41, 8692–8705, doi: 10.1093/nar/gkt619 (2013). 23863838 PMC3794582

[b18] Coller J. & Parker R. General translational repression by activators of mRNA decapping. Cell 122, 875–886, doi: 10.1016/j.cell.2005.07.012 (2005). 16179257 PMC1853273

[b19] Eulalio A., Behm-Ansmant I., Schweizer D. & Izaurralde E. P-body formation is a consequence, not the cause, of RNA-mediated gene silencing. Mol Cell Biol 27, 3970–3981, doi: 10.1128/MCB.00128-07 (2007). 17403906 PMC1900022

[b20] Ayache J. . P-body assembly requires DDX6 repression complexes rather than decay or Ataxin2/2L complexes. Mol Biol Cell 26, 2579–2595, doi: 10.1091/mbc.E15-03-0136 (2015). 25995375 PMC4501357

[b21] Kruk J. A., Dutta A., Fu J., Gilmour D. S. & Reese J. C. The multifunctional Ccr4-Not complex directly promotes transcription elongation. Genes Dev 25, 581–593, doi: 10.1101/gad.2020911 (2011). 21406554 PMC3059832

[b22] Haimovich G. . Gene expression is circular: factors for mRNA degradation also foster mRNA synthesis. Cell 153, 1000–1011, doi: 10.1016/j.cell.2013.05.012 (2013). 23706738

[b23] Yoshida-Kashikawa M., Shibata N., Takechi K. & Agata K. DjCBC-1, a conserved DEAD box RNA helicase of the RCK/p54/Me31B family, is a component of RNA-protein complexes in planarian stem cells and neurons. Dev Dynam 236, 3436–3450, doi: 10.1002/dvdy.21375 (2007). 17994545

[b24] Smillie D. A. & Sommerville J. RNA helicase p54 (DDX6) is a shuttling protein involved in nuclear assembly of stored mRNP particles. J Cell Sci 115, 395–407 (2002). 11839790 10.1242/jcs.115.2.395

[b25] Kawahara C., Yokota S. & Fujita H. DDX6 localizes to nuage structures and the annulus of mammalian spermatogenic cells. Histochem Cell Biol 141, 111–121, doi: 10.1007/s00418-013-1153-2 (2014). 24141902

[b26] Iio A. . DDX6 post-transcriptionally down-regulates miR-143/145 expression through host gene NCR143/145 in cancer cells. Biochim Biophys Acta 1829, 1102–1110, doi: 10.1016/j.bbagrm.2013.07.010 (2013). 23932921

[b27] Pettersson O. J. . DDX6 regulates sequestered nuclear CUG-expanded DMPK-mRNA in dystrophia myotonica type 1. Nucleic Acids Res 42, 7186–7200, doi: 10.1093/nar/gku352 (2014). 24792155 PMC4066779

[b28] Marnef A., Weil D. & Standart N. RNA-related nuclear functions of human Pat1b, the P-body mRNA decay factor. Mol Biol Cell 23, 213–224, doi: 10.1091/mbc.E11-05-0415 (2012). 22090346 PMC3248899

[b29] Nishi K., Nishi A., Nagasawa T. & Ui-Tei K. Human TNRC6A is an Argonaute-navigator protein for microRNA-mediated gene silencing in the nucleus. RNA 19, 17–35, doi: 10.1261/rna.034769.112 (2013). 23150874 PMC3527724

[b30] Ernoult-Lange M. . Nucleocytoplasmic traffic of CPEB1 and accumulation in Crm1 nucleolar bodies. Mol Biol Cell 20, 176–187, doi: 10.1091/mbc.E08-09-0904 (2009). 18923137 PMC2613105

[b31] Gagnon K. T., Li L. D., Janowski B. A. & Corey D. R. Analysis of nuclear RNA interference in human cells by subcellular fractionation and Argonaute loading. Nat Protoc 9, 2045–2060, doi: 10.1038/nprot.2014.135 (2014). 25079428 PMC4251768

[b32] Gagnon K. T., Li L. D., Chu Y. J., Janowski B. A. & Corey D. R. RNAi Factors Are Present and Active in Human Cell Nuclei. Cell Rep 6, 211–221, doi: 10.1016/j.celrep.2013.12.013 (2014). 24388755 PMC3916906

[b33] Kirli K. . A deep proteomics perspective on CRM1-mediated nuclear export and nucleocytoplasmic partitioning. eLife 4, doi: 10.7554/eLife.11466 (2015). PMC4764573 26673895

[b34] Matsui T. . Structural insight of human DEAD-box protein rck/p54 into its substrate recognition with conformational changes. Genes to cells 11, 439–452, doi: 10.1111/j.1365-2443.2006.00951.x (2006). 16611246

[b35] la Cour T. . Analysis and prediction of leucine-rich nuclear export signals. Protein Eng Des Sel 17, 527–536, doi: 10.1093/protein/gzh062 (2004). 15314210

[b36] Brameier M., Krings A. & MacCallum R. M. NucPred--predicting nuclear localization of proteins. Bioinformatics 23, 1159–1160, doi: 10.1093/bioinformatics/btm066 (2007). 17332022

[b37] Marfori M., Lonhienne T. G., Forwood J. K. & Kobe B. Structural basis of high-affinity nuclear localization signal interactions with importin-alpha. Traffic 13, 532–548, doi: 10.1111/j.1600-0854.2012.01329.x (2012). 22248489

[b38] Kataoka N. . Specific Y14 domains mediate its nucleo-cytoplasmic shuttling and association with spliced mRNA. Sci Rep 1, 92, doi: 10.1038/srep00092 (2011). 22355610 PMC3216578

[b39] Kataoka N., Dobashi I., Hagiwara M. & Ohno M. hDbr1 is a nucleocytoplasmic shuttling protein with a protein phosphatase-like motif essential for debranching activity. Sci Rep 3, 1090, doi: 10.1038/srep01090 (2013). 23346348 PMC3549538

[b40] Ozgur S. . Structure of a Human 4E-T/DDX6/CNOT1 Complex Reveals the Different Interplay of DDX6-Binding Proteins with the CCR4-NOT Complex. Cell Rep 13, 703–711, doi: 10.1016/j.celrep.2015.09.033 (2015). 26489469

[b41] Kuzuoglu-Ozturk D. . miRISC and the CCR4-NOT complex silence mRNA targets independently of 43S ribosomal scanning. EMBO J 35, 1186–1203, doi: 10.15252/embj.201592901 (2016). 27009120 PMC4888236

[b42] Ohta S. . The protein composition of mitotic chromosomes determined using multiclassifier combinatorial proteomics. Cell 142, 810–821, doi: 10.1016/j.cell.2010.07.047 (2010). 20813266 PMC2982257

[b43] Torrente M. P. . Proteomic interrogation of human chromatin. PLoS ONE 6, e24747, doi: 10.1371/journal.pone.0024747 (2011). 21935452 PMC3173473

[b44] Dostie J., Ferraiuolo M., Pause A., Adam S. A. & Sonenberg N. A novel shuttling protein, 4E-T, mediates the nuclear import of the mRNA 5′ cap-binding protein, eIF4E. EMBO J 19, 3142–3156, doi: 10.1093/emboj/19.12.3142 (2000). 10856257 PMC203362

[b45] Kolker E. . MOPED: Model Organism Protein Expression Database. Nucleic Acids Res 40, D1093–1099, doi: 10.1093/nar/gkr1177 (2012). 22139914 PMC3245040

[b46] Nagaraj N. . Deep proteome and transcriptome mapping of a human cancer cell line. Mol Syst Biol 7, 548, doi: 10.1038/msb.2011.81 (2011). 22068331 PMC3261714

[b47] Wuhr M. . Deep proteomics of the Xenopus laevis egg using an mRNA-derived reference database. Curr Biol 24, 1467–1475, doi: 10.1016/j.cub.2014.05.044 (2014). 24954049 PMC4090281

[b48] Kamenska A. . The DDX6-4E-T interaction mediates translational repression and P-body assembly. Nucleic Acids Res, doi: 10.1093/nar/gkw565 (2016). PMC5291280 27342281

[b49] Minshall N., Thom G. & Standart N. A conserved role of a DEAD box helicase in mRNA masking. RNA 7, 1728–1742 (2001). 11780630 10.1017/s135583820101158x PMC1370213

[b50] Cheng Z., Coller J., Parker R. & Song H. Crystal structure and functional analysis of DEAD-box protein Dhh1p. RNA 11, 1258–1270, doi: 10.1261/rna.2920905 (2005). 15987810 PMC1370809

[b51] Dutta A., Zheng S. T., Jain D., Cameron C. E. & Reese J. C. Intermolecular interactions within the abundant DEAD-box protein Dhh1 regulate its activity *in vivo*. J Biol Chem 286, 27454–27470, doi: 10.1074/jbc.M111.220251 (2011). 21642421 PMC3149339

[b52] Ernoult-Lange M. . Multiple binding of repressed mRNAs by the P-body protein Rck/p54. RNA 18, 1702–1715, doi: 10.1261/rna.034314.112 (2012). 22836354 PMC3425784

[b53] Mitchell S. F., Jain S., She M. P. & Parker R. Global analysis of yeast mRNPs. Nat Struct Mol Biol 20, 127–U161, doi: 10.1038/nsmb.2468 (2013). 23222640 PMC3537908

[b54] de Vries S. . Identification of DEAD-box RNA helicase 6 (DDX6) as a cellular modulator of vascular endothelial growth factor expression under hypoxia. J Biol Chem 288, 5815–5827, doi: 10.1074/jbc.M112.420711 (2013). 23293030 PMC3581395

[b55] Saito K., Kondo E. & Matsushita M. MicroRNA 130 family regulates the hypoxia response signal through the P-body protein DDX6. Nucleic Acids Res 39, 6086–6099, doi: 10.1093/nar/gkr194 (2011). 21486751 PMC3152344

[b56] Yoon J. H. . LincRNA-p21 suppresses target mRNA translation. Mol Cell 50, 303–303, doi: 10.1016/j.molcel.2013.04.008 (2013). PMC3509343 22841487

[b57] Muck F., Bracharz S. & Marschalek R. DDX6 transfers P-TEFb kinase to the AF4/AF4N (AFF1) super elongation complex. Am J Blood Res 6, 28–45 (2016). 27679741 PMC5030405

[b58] Derrien T. . The GENCODE v7 catalog of human long noncoding RNAs: Analysis of their gene structure, evolution, and expression. Genome Res 22, 1775–1789, doi: 10.1101/gr.132159.111 (2012). 22955988 PMC3431493

[b59] Cabili M. N. . Localization and abundance analysis of human IncRNAs at single-cell and single-molecule resolution. Genome Biol 16, doi: ARTN 2010.1186/s13059-015-0586-4 (2015). 10.1186/s13059-015-0586-4 PMC4369099 25630241

[b60] Ladomery M. & Sommerville J. The Scd6/Lsm14 protein xRAPB has properties different from RAP55 in selecting mRNA for early translation or intracellular distribution in Xenopus oocytes. Biochim Biophys Acta 1849, 1363–1373, doi: 10.1016/j.bbagrm.2015.10.002 (2015). 26455898

[b61] Feng L. . Discovery of a small-molecule BMP sensitizer for human embryonic stem cell differentiation. Cell Rep 15, 2063–2075, doi: 10.1016/j.celrep.2016.04.066 (2016). 27210748 PMC4889468

[b62] Boersema P. J., Raijmakers R., Lemeer S., Mohammed S. & Heck A. J. R. Multiplex peptide stable isotope dimethyl labeling for quantitative proteomics. Nat Protoc 4, 484–494, doi: 10.1038/nprot.2009.21 (2009). 19300442

[b63] Rappsilber J., Mann M. & Ishihama Y. Protocol for micro-purification, enrichment, pre-fractionation and storage of peptides for proteomics using StageTips. Nat Protoc 2, 1896–1906, doi: 10.1038/nprot.2007.261 (2007). 17703201

[b64] Cox J. & Mann M. MaxQuant enables high peptide identification rates, individualized p.p.b.-range mass accuracies and proteome-wide protein quantification. Nat Biotechnol 26, 1367–1372, doi: 10.1038/nbt.1511 (2008). 19029910

