# Supplementary material for: Dual mechanisms regulate the nucleocytoplasmic localization of human DDX6
Source: Sci Rep. 2017 Feb 20;7:42853. doi: 10.1038/srep42853 (PMC5316971; doi:10.1038/srep42853)
Supplement: Supplementary Information [file srep42853-s1.pdf]

Supplementary Information

Dual mechanisms regulate the nucleocytoplasmic localization of human DDX6

Jo-Hsi Huang[1], Wei-Chi Ku[2], Yen-Chun Chen[1], Yi-Ling Chang[2], and Chia-Ying Chu[1, 3]*

[1] Department of Life Science, College of Life Science, National Taiwan University, Taipei 10617, Taiwan.

[2] School of Medicine, College of Medicine, Fu Jen Catholic University, New Taipei 24205, Taiwan.

[3] Center for Systems Biology, National Taiwan University, Taipei 10617, Taiwan.

Supplementary Table S1. DDX6 peptides in HeLa proteome identified by mass spectrometry

Peptide Sequence	Length (AA)	Position in Protein	Peptide Score*	Cytoplasmic (Rep. 1)	Cytoplasmic (Rep. 2)	Nuclear (Rep. 1)	Nuclear (Rep. 2)
DNIQAMVIVPTR	12	163 - 174	111.1		v		v
GNEFEDYCLK	10	95 - 104	86.5				v
GPVKPTGGPGGGGTQTQQQMNQLK	24	23 - 46	139.4		v		v
GVTQYYAYVTER	12	308 -319	95.3			v	v
LAETYLHR	8	414 -421	69.2			v	
LDDTVHVVIATPGR	14	212 - 225	94.7				v
NLVCTDLFTR	10	387 - 396	60.7	v		v	v
SGAYLIPLLER	11	147 - 157	90.6			v	v
SIEEQLGTEIKPIPSNIDK	19	448 - 466	113.5				v
SLYVAEYHSEPVEDEKP	17	467 - 483	105.2	v	v	v	v
VMATTGGTNLR	11	196 - 206	26.8		v		v

Supplementary Table S2. List of the siRNA

Name	Target gene	Target sequence (sense; 5' – 3')
siDDX6-mm [a]	DDX6 (Mis-matched)	GCAGA AACGG UAUGA GAUU
siDDX6-pm	DDX6 (Perfectly matched)	GCAGA AACCC UAUGA GAUU
siControl [b]	Non-targeting to human genes	UAGCG ACUAA ACACA UCAA
siAGO2	AGO2	GCACG GAAGU CCAUC UGAA
siMALAT1-N	MALAT1	GAAAG CGAGU GGUUG GUAA

a, Control for DDX6 knockdown.

b, Control for AGO2 and MALAT1 knockdown.

For 4E-T knockdown, we used siGENOME Human EIF4ENIF1 (56478) SMARTpool siRNA (M-013237-00-0005, Dharmacon).

Supplementary Table S3. List of qPCR primers

Gene	Directionality	Sequence (5'-3')
GAPDH	F	CCA TGA GAA GTA TGA CAA CAG CC
	R	GGG TGC TAA GCA GTT GGT G
DDX6	F	GCT GGG AAA AGC CAT CTC CTA
	R	GGT CTA GCC GTT CAA GTA AGG G
MALAT1	F	GAA TGC TTG AAG TAC CCC TGG
	R	CTG GAA AAG CTA GGG AAA AGT GG
NEAT1	F	ACA GAG TCA CCA GTT TTC CG
	R	ACT TGA TAA CAC CCA CAC CC
MYC	F	CCT GGT GCT CCA TGA GGA GAC
	R	CAG ACT CTG ACC TTT TGC CAG G
AGO2	F	CGG AAG CCC GTG TTT GAC GG
	R	CGC AGG ACA CCC ACT TGA TGG
GAS5	F	CTT GCC TGG ACC AGC TTA AT
	R	CAA GCC GAC TCT CCA TAC CT

Supplementary Figure S1. Assessment of the WB detection of nuclear DDX6

(a) Representative WB of fractionation following DDX6 KD by various siRNA concentrations. **(b)** Densitometric quantification of the WB results. Points and error bars represent means and standard errors of the mean over three biological replicates, respectively. **(c)** Representative WB showing the comparison of different methods to equalize input amounts for SDS-PAGE. Equalizing by protein mass means the same amount of nuclear and cytoplasmic extracts (20 μg) were loaded side-by-side, whereas equalizing by cell numbers means the nuclear and cytoplasmic extracts prepared from the same number of cells (1×10^5 cells) were loaded side-by-side.

Supplementary Figure S2. IF detects endogenous DDX6 in HeLa cell nuclei.

(a) Illustration represents the spatial settings of confocal image acquisition. A total of 11 optical slices were captured across a 10 μm range along the Z axis (cell height) with 1 μm spacing. The resulting image stack was projected 3-dimensionally. **(b)** IF detects endogenous DDX6 in HeLa nuclei. The two cells were cropped, projected 3-dimesionally, and rotated to show nuclear staining of DDX6. Grid lines in X- and Z- axis represent 2 μm interval, and represent 5 μm spacing in Y axis.

Supplementary Figure S3. Assessment of the IF detection of nuclear DDX6

(a) Schematic illustration of the workflow for assessing IF detection of nuclear DDX6. Cell transfected with siDDX6-mm or co-transfected with siDDX6-pm and mCherry were co-cultured prior to IF and imaging. **(b)** Representative images of anti-DDX6 IF and control experiment without primary antibody. **(b, i and iv)** Merged images of the three channels. **(b, ii and v)** Images showing LUT of anti-DDX6 IF signals. **(b, iii and vi)** Images showing overlay of anti-DDX6 IF and nuclear masks. Scale bars represent 10 μm. **(c-e)** Quantification of the mean IF signal within the nuclear regions. **(c)** Quantification of each section along the Z-axis of the two representative cells shown in **(b, i-iii)**. The nuclear masks were determined for each section by Hoechst staining. Each point represents quantification of one Z-section. N = 15 sections. **(d)** Quantification of the nuclear DDX6 signal in different cells from two replicates. Images were obtained from 3 (Rep. 2) or 4 (Rep. 1) fields under confocal microscope. Each dot represents a measured cell. $N_{siDDX6\text{-}mm}$ = 14 and 8 cells for Rep. 1 and

2, respectively. $N_{siDDX6\text{-}pm}$ = 19 and 14 cells for Rep. 1 and 2, respectively. **(e)** Quantification of the nuclear DDX6 signal in different cells from control IF experiment without primary antibody. $N_{siDDX6\text{-}mm}$ = 5 and $N_{siDDX6\text{-}pm}$ = 26. **(c-e)** Mid-lines represent means of nuclear anti-DDX6 IF signal. Error bars represent standard deviations. **(b-e)** Images were taken with the same microscopic setting. To enable cross-image comparison for anti-DDX6 IF signal, no post-acquisition adjustment was made to the anti-DDX6 IF signals.

Supplementary Figure S4. Imaging nuclear YFP-DDX6

(a) Merged image of a representative HeLa cell expressing YFP-DDX6 FL (green) and stained with Hoechst (blue). The white dashed line represents the pixels measured for RGB profile as shown in **(b)**, and the arrow represents the direction of measurement. **(c)** The LUT of YFP-DDX6 FL signal. **(d)** Three randomly selected square areas in the nuclear region and in the nearby irrelevant region (background) were measured for YFP signals as shown in **(e)**. Scale bar = 10 μm. (e) Bar chart showing quantification of the areas selected in **(d)**. Bar height represents mean and error bar represents standard deviation.

Supplementary Figure S5. DDX6 binds to the nuclear lncRNA MALAT1.

(a) MALAT1 is co-IPed with DDX6. WB shows that DDX6 is selectively IPed by anti-DDX6 antibody but not by control IgG. Relative abundance of RNA co-IPed with DDX6 was assessed with RT-qPCR. MYC mRNA serves as the positive control for anti-DDX6 co-IP. Bar-heights and Error bars represent means and standard deviations of fold enrichments (anti-DDX6/IgG) calculated from 6 independent experiments, respectively. Dashed line represent ratio = 1. P values were calculated from right-tailed one-sample t test. Both MYC and MALAT1 enrichment are significant at α = 0.05. **(b) DDX6 co-localizes with MALAT1 *in vivo*.** Co-localization between MALAT1 (shown in red) and DDX6 (shown in green) in the nuclei was tracked by confocal microscopy following FISH and IF double staining. A single optical section of one representative nucleus is shown. Insets display details of DDX6 co-localizing with, and docking to, MALAT1. Nucleus was counter-stained by Hoechst (shown in blue). Scale bar = 5 μm.

Supplementary Figure S6. Assessment of the interaction between DDX6 and MALAT1

(a) The WB of total cell extract and anti-DDX6 IP eluent. Anti-DDX6 IP was performed with both control (siDDX6-mm) and DDX6-depleted cells (siDDX6-pm). IP experiments were carried out using either control IgG or DDX6-specific antibody (A300-460A, Bethyl Laboratory). 4E-T, DCP1A, and CRM1 are known PPI partners of DDX6. **(b)** Anti-DDX6 RIP-qPCR of MALAT1 in control (siDDX6-mm) and DDX6-depleted cells (siDDX6-pm). Bar height and error bars represents mean and standard deviation summarized from four repeat experiments. **(c)** MALAT1 FISH (red) and phosphorylated SC35 (p-SC35; green) IF double staining in HeLa cells. **(d)** DDX6 (green) and p-SC35 (red) double IF staining. (c and d) The nuclei were stained with Hoechst (blue). Scale bar = 5 μm.

Supplementary Figure S7. Fractionation and WB following LMB treatment

Two replicated experiments of WB following LMB treatment and fractionation. The HeLa cells were subjected to a range of LMB doses from 5 to 50 ng/mL as in Fig. 2b.

Supplementary Figure S8. Visualization of the putative NES from two published DDX6 structures.

The structure of DDX6 NTD (4CT4 and 1VEC) was visualized. Cylinders represent α-helices; flat arrows represent β-sheets. NES peptide backbone is colored in red; carbon atoms of leucine residues are colored in green. Insets display magnified views of the boxed areas near the putative NES.

Supplementary Figure S9. The putative NES is stably folded in the NTD of DDX6 and may serve a structure role.

(a) The putative NES is stably folded within the 3 published structures. The B factor spectrum of the NTD of DDX6 in three different published structures. The B factors from the three structures were standardized to give comparable values. The colored area represents the putative NES segment. Since B factor can be interpreted as an index of local structural flexibility, the relative low B factor of the putative NES indicates relatively stable folding with DDX6 NTD. **(b)** The jitter plot

showing the comparison between real mean B factors and the permutation results. The mean B factors were taken over a sliding window of 10 AA. For each position, the B factors were given by the mean of the standardized values (Z score) from the three crystal structures. The real data were plot as points. Each point represents one window. The randomly permuted data (100 permutations, 19600 windows) were summarized was the grey distribution, representing the null distribution. P value was estimated from this null distribution. The putative NES has $p = 7.15 \times 10^{-4}$. **(c)** Sequence alignment of the putative NES region of DDX6 and the homologous regions in several human DBPs of the eIF4A-like clade. **(d)** The bar graphs showing the enrichment of the hydrophobic AA in the homologous segments of the putative NES in other eIF4A-like DBPs. The enrichment ratios were calculated using FL protein sequences as backgrounds. The dashed lines represent no enrichment. **(e)** The contingency tables for the hydrophobic AA in the homologous segments of the putative NES in other eIF4A-like DBPs. Three representative tables were shown, including the eIF4A-like DBPs as a whole, DDX6, and DDX2A. P values were calculated using the Fisher exact test.

Supplementary Figure S10. K/R-rich NLS does not facilitate DDX6 nuclear localization.

(a) Illustration depicts the YFP-DDX6 1-166 WT and mNLS. The blue segment represents the putative K/R-rich NLS of DDX6. Sequence alignment of the human, the mouse, and the *Xenopus* DDX6 putative NLS was shown. Asterisk denotes residue targeted for mutagenesis (K73 and K74; K to A substitution). Red segment: the putative NES. **(b) Nuclear entry of YFP-DDX6 1-166 is not abolished by NLS mutation.** HeLa cells expressing YFP-4E-T, YFP-DDX6 1-166 WT and mNLS were treated with 5 ng/mL LMB for 5 hr prior to imaging. YFP signal is shown in green. Nuclei were stained with Hoechst (shown in blue). Scale bar = 20 μm. **(c)** Prediction by NucPred does not report potential K/R-rich NLS target in DDX6 sequence. Potential NLS reported by NucPred should be colored in yellow/orange/red and highlighted by underlining key residues, as is shown for the nuclear-localized DRB1 CTD that was used in chimeric protein assay (Fig. 4c and d).

Supplementary Figure S11. Pharmacological assessment of the putative NLS of DDX6

(a) Representative WB of fractionation following importazole (IPZ) treatment by various

concentrations. **(b)** Densitometric quantification of the WB results. Points and error bars represent means and standard errors of the mean over three biological replicates, respectively. **(c)** Nuclear import assay of YFP-DDX6 1-166. HeLa cells expressing YFP-DDX6 1-166 or YFP-4E-T were first treatment with IPZ (80 μM) for 3 hours prior to subsequent addition of LMB (5 ng/mL) for another 2 hr. Solvents for IPZ and LMB, namely DMSO and 70% methanol, were used as negative controls. The YFP signals were shown in green. Nuclei were stained with Hoechst (blue). Scale bars = 20 μm.

Supplementary Figure S12. RISC and MALAT1 are not required for the nuclear entry of DDX6.

(a) AGO2 knockdown does not alter DDX6 expression level. AGO2 and DDX6 expression levels were assessed with qPCR and WB. **(b)** AGO2 knockdown does not alter DDX6 level in nuclear and cytoplasmic extracts. AGO2 and DDX6 protein levels were assessed with WB **(c)** MALAT1 knockdown does not alter DDX6 expression level. MALAT1 and DDX6 RNA levels were assessed by qPCR. DDX6 protein level was assessed with WB. **(d)** MALAT1 knockdown does not alter DDX6 level in nuclear and cytoplasmic extracts. DDX6 protein level was assessed with WB. **(a and c)** HeLa cell transfected with control or targeting siRNA were harvested 48 hours post-transfection. GAPDH mRNA was used as internal control in qPCR. Bar heights and error bars represent means and standard deviations of relative expression levels calculated from 3 biological replicates. GAPDH protein was used as loading control for WB. **(b and d)** HeLa cell transfected with control or targeting siRNA were fractionated with NE-PER reagents 48 hours post-transfection. Equal amount of nuclear and cytoplasmic extracts were separated side-by-side with SDS-PAGE and then analyzed with WB. LMNA: nuclear marker; GAPDH: cytoplasmic marker.

Supplementary Figure S13. The DDX6 CTD localizes to the cell nuclei.

(a) The diagram representing the DDX6 constructs used in this figure. **(b)** The representative WB showing detection of YFP-DDX6 constructs in the HeLa cytoplasmic and the nuclear extracts. LMNA: nuclear marker; GAPDH: cytoplasmic marker. **(c)** The representative images of YFP- and myc-DDX6 CTD. **(d)** The representative images of the live and the fixed HeLa cells expressing YFP-DDX6 CTD. **(c and d)**, YFP and anti-myc IF signals were shown in green. The nuclei were stained with Hoechst

(shown in blue). Scale bar = 20 μm.

Supplementary Figure S14. DDX6 CTD nuclear localization is not abolished by mutations in protein binding sites, conserved helicase motifs, and consensus phosphorylation sites.

(a) Illustration depicts protein binding sites, conserved helicase motifs, and consensus phosphorylation sites. White box: DDX6 CTD. Brown segment: protein binding site. Pink segment: helicase motif. Purple segment: phosphorylation site. Patch 2: EDC3 binding site; patch 3: 4E-T and PATL1 binding site; R386: CNOT1 binding site. PS: phosphorylation site. Consensus PS in DDX6 CTD is defined as residue with more than 10 HTP records in PhosphoSitePlus database (accessed in October, 2015). **(b-d)** Mutations in protein binding sites, helicase motifs and phosphorylation sites do not abolish DDX6 CTD nuclear localization. HeLa cells expressing YFP-DDX6 CTD WT and mutants were imaged live. YFP signal is shown in green. Nuclei were stained with Hoechst (shown in blue). Scale bar = 20 μm. **(b)** Mut1 (Q320A, H323A, T327A, R331A), mut2 (R443A, F444A, K447A, E450A, E451) and mut3 (S343D, Q345D, S346D) represent patch 1, 2, and 3 mutant, respectively. **(c)** mMotif: helicase motif mutant. **(d)** mPS: phosphorylation site mutant. DDX6 CTD mPS and mMotif mutants bear AA substitution to alanine.

Supplementary Figure S15. Construction and expression of YFP-DDX6 Rec.

(a) YFP-DDX6 Rec was constructed by inserting DDX6 CTD DNA fragment to the 3' end of YFP-DDX6 NTD with SalI site, the same linker used for generating chimeric YFP-PKM-DDX6 constructs. Rec: reconstituted. DNA and protein sequence alignments of the linker region of DDX6 FL and DDX6 Rec are shown. **(b and c)** Subcellular localization of YFP-DDX6 FL and -Rec in interphase and prometaphase HeLa cells. Interphase cells were imaged from asynchronous HeLa cell population. Prometaphase cells were imaged from synchronized population. YFP signal is shown in green. Nuclei and mitotic chromosomes were stained with Hoechst (shown in blue). Scale bar = 20 μm (interphase cells) or 10 μm (prometaphase cells). Granule +: granule-positive phenotype. Double +: granule/chromosome double-positive phenotype. Chr. +: chromosome-positive phenotype. Numbers represent means and standard deviations of the percentage of the displayed phenotypes

calculated from 3 biological replicates.

Supplementary Figure S16. DDX6 generally outnumbers its shuttling protein partners in abundance.

(a-e) The bar graphs and the violin plots showing the protein stoichiometry of the DDX6 homologs with their protein partners. The quantitative MS results of the stage VI Xenopus oocyte cytoplasm **(a)**, the nuclei **(b)**, the unfertilized eggs **(c)**, the HeLa cells **(d)**, the various human cell lines **(e)**, and various human tissues **(f)** were plotted. CNOT1, EDC3, LSM14A are not transported by the CRM1 pathway *per se*. In contrast, 4E-T, PATL1, and LSM14B are known cargoes for the CRM1 pathway (Fig. 2 and Kirli et al., 2015). The quantitative MS data were collected from Kirki et al., 2015 **(a and b)**, Wuhr et al., 2014 **(c)**, Nagaraj et al., 2011 **(d)**, and the MOPED database **(e and f)**.

Supplementary Figure S17. Fractionation and WB following 4E-T KD

The representative WB of fractionation following 4E-T knockdown. EIF4E is a known transport cargo of 4E-T. LMNA was used as marker for nuclear fraction. GAPDH was used as a marker for the cytosolic fraction. CALR was used as a marker for ER.

Supplementary Figure S18. Anti-DDX6 IF in mitotic HeLa cells and its assessment

(a) Anti-DDX6 IF (shown in green) in mitotic HeLa cells with three different antibodies. 1: anti-DDX6 (A300-460A, Bethyl Laboratory); 2: anti-DDX6 (A300-461A, Bethyl Laboratory); 3: anti-DDX6 (sc-51415, Santa Cruz). Mitotic chromosomes were stained with Hoechst (shown in blue). **(b)** Anti-DDX6 IF in mitotic DDX6-depleted HeLa cells. Control (siDDX6-mm) and DDX6-specific (siDDX6-pm) siRNAs were co-transfected with mCherry. Scale bars = 5 μm. **(b, i and iv)** Merged images of anti-DDX6 IF (A300-460A, Bethyl Laboratory; shown in green), mitotic chromosomes (stained with Hoechst; shown in blue), and mCherry (red) were shown. **(b, ii and v)** The LUT of anti-DDX6 IF signals. **(b, iii and vi)** The anti-DDX6 IF signals. **(c)** Quantification of the mean anti-DDX6 IF signal across the optical section for each cells. To this end, ellipses were fitted to each measured cells. Each dot represents one measured cell. Mid-line and error bar represent mean and standard deviation, respectively. $N_{siDDX6\text{-}mm}$ = 16. $N_{siDDX6\text{-}pm}$ = 9. To enable cross-image comparison of

the anti-DDX6 IF signals, no post-acquisition adjustment were made to the anti-DDX6 IF signals. **(d)** Effects of fixation by 4% paraformaldehyde of the subnuclear localization of YFP-DDX6 CTD. The YFP signal is shown in green. The nuclei were stained with Hoechst and are shown in blue. Scale bars = 10 μm.

Supplementary Figure S19. Assessment of the effects of cycloheximide and actinomycin D on the nucleocytoplasmic distribution of DDX6

(a and b) Assessing the effect of actinomycin D (AMD) on the nucleocytoplasmic localization of DDX6. The HeLa cells expressing YFP-DDX6 FL **(a)** or untransfected HeLa cells **(b)** were treated with 5 μg/mL AMD for 2 hours prior to imaging **(a)** or fractionation followed by WB **(b)**. DMSO, the solvent for AMD, was used as control. **(c and d)** Assessing the effect of cycloheximide (CHX) on the nucleocytoplasmic localization of DDX6. The HeLa cells expressing YFP-DDX6 FL **(c)** or untransfected HeLa cells **(d)** were treated with 100 μg/mL CHX for 2 hours prior to imaging **(c)** or fractionation followed by WB **(d)**. PBS, the solvent for CHX, was used as control. **(a and c)** The YFP signal is shown in green. Nuclei were stained with Hoechst and are shown in blue. Scale bars = 20 μm. **(b and d)** Equal amount (protein mass) of the cytoplasmic and nuclear extracts were loaded side-by-side for WB.

Supplementary Figure S20. The nucleocytoplasmic distribution of full length DDX6 mutants

(a-c) The localization of the YFP-DDX6 FL protein-binding sites **(a)**, conserved helicase motifs **(b)**, and consensus phosphorylation site **(c)** mutants. The mutants are the same as described in Fig. S14 albeit introduced to DDX6 FL. YFP signals are shown in green. The nuclei were stained with Hoechst (blue). Scale bars = 20 μm.

Fig. S1

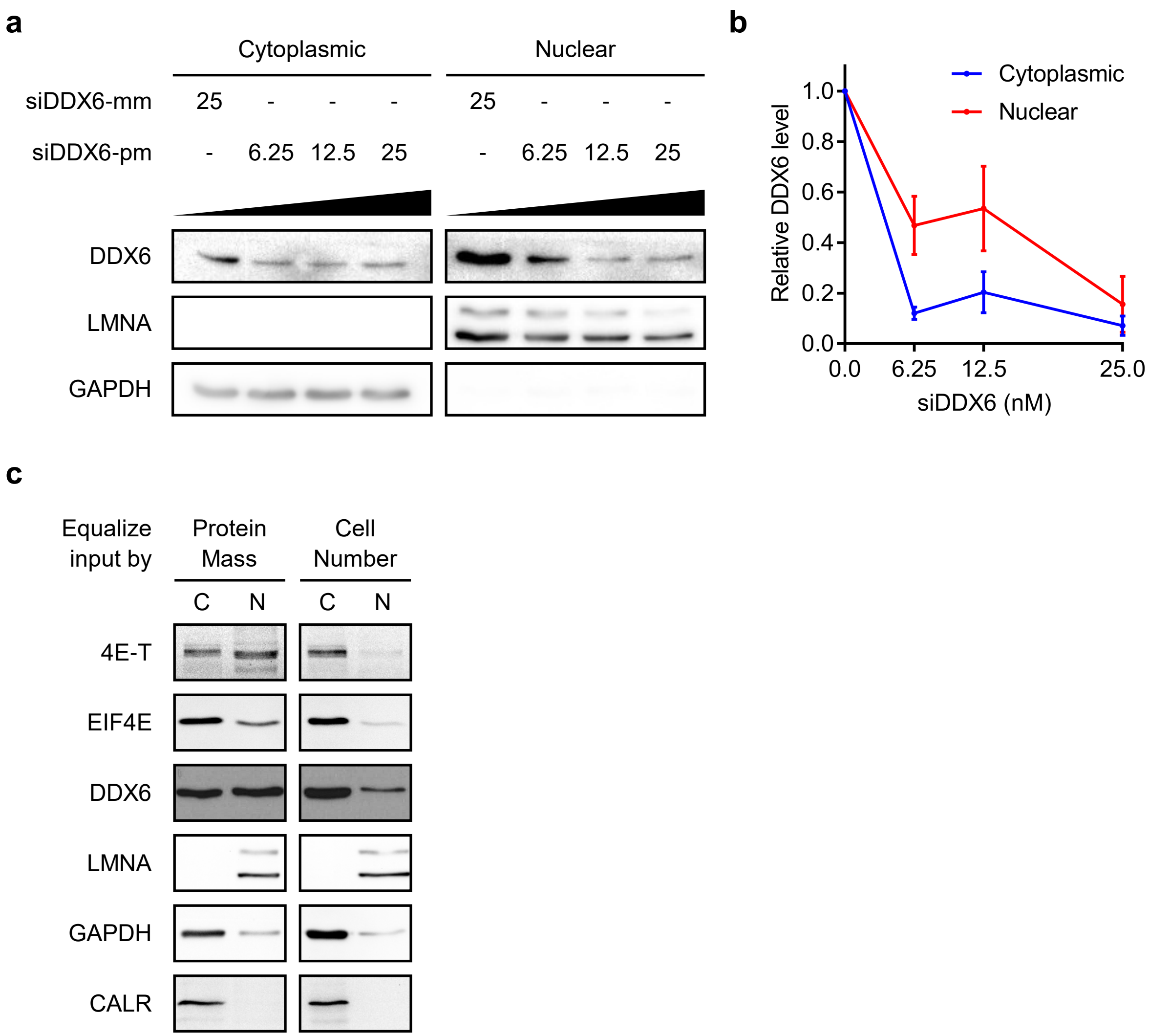
a
Cytoplasmic
Nuclear
siDDX6-mm
25
-
-
-
25
-
-
-
siDDX6-pm
-
6.25
12.5
25
-
6.25
12.5
25
DDX6
LMNA
GAPDH
b
Cytoplasmic
Nuclear
Relative DDX6 level
1.0
0.8
0.6
0.4
0.2
0.0
0.0
6.25
12.5
25.0
siDDX6 (nM)
c
Equalize input by
Protein Mass
Cell Number
C
N
C
N
4E-T
EIF4E
DDX6
LMNA
GAPDH
CALR

Fig. S2

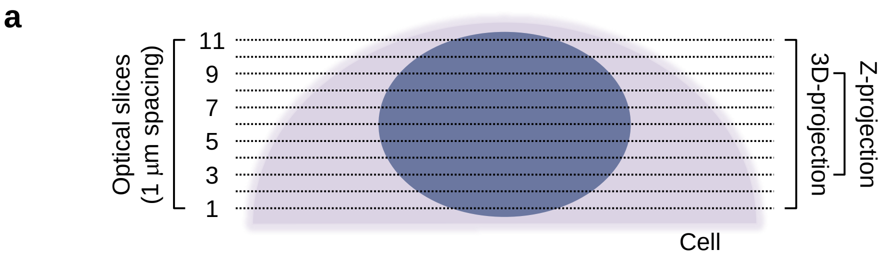

a
Optical slices
(1 μm spacing)
11
9
7
5
3
1
3D-projection
Z-projection
Cell

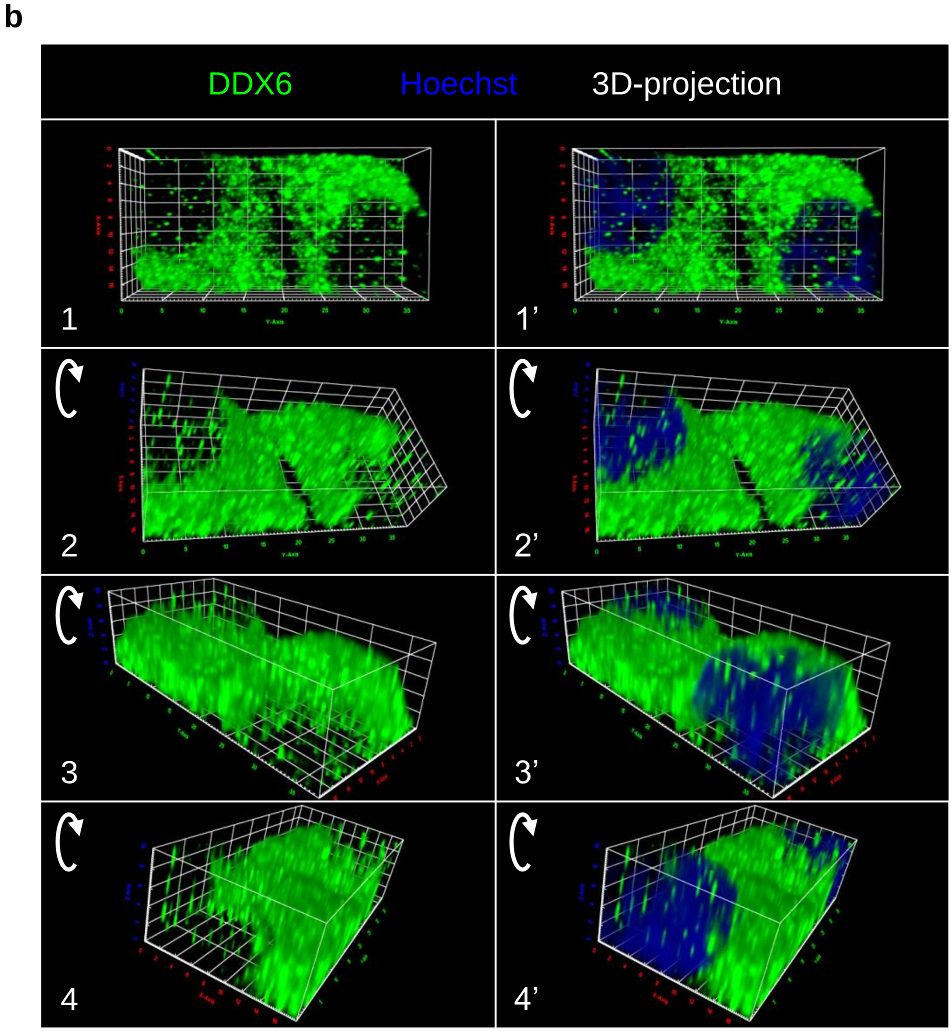

b
DDX6
Hoechst
3D-projection
1
1'
2
2'
3
3'
4
4'

Fig. S3

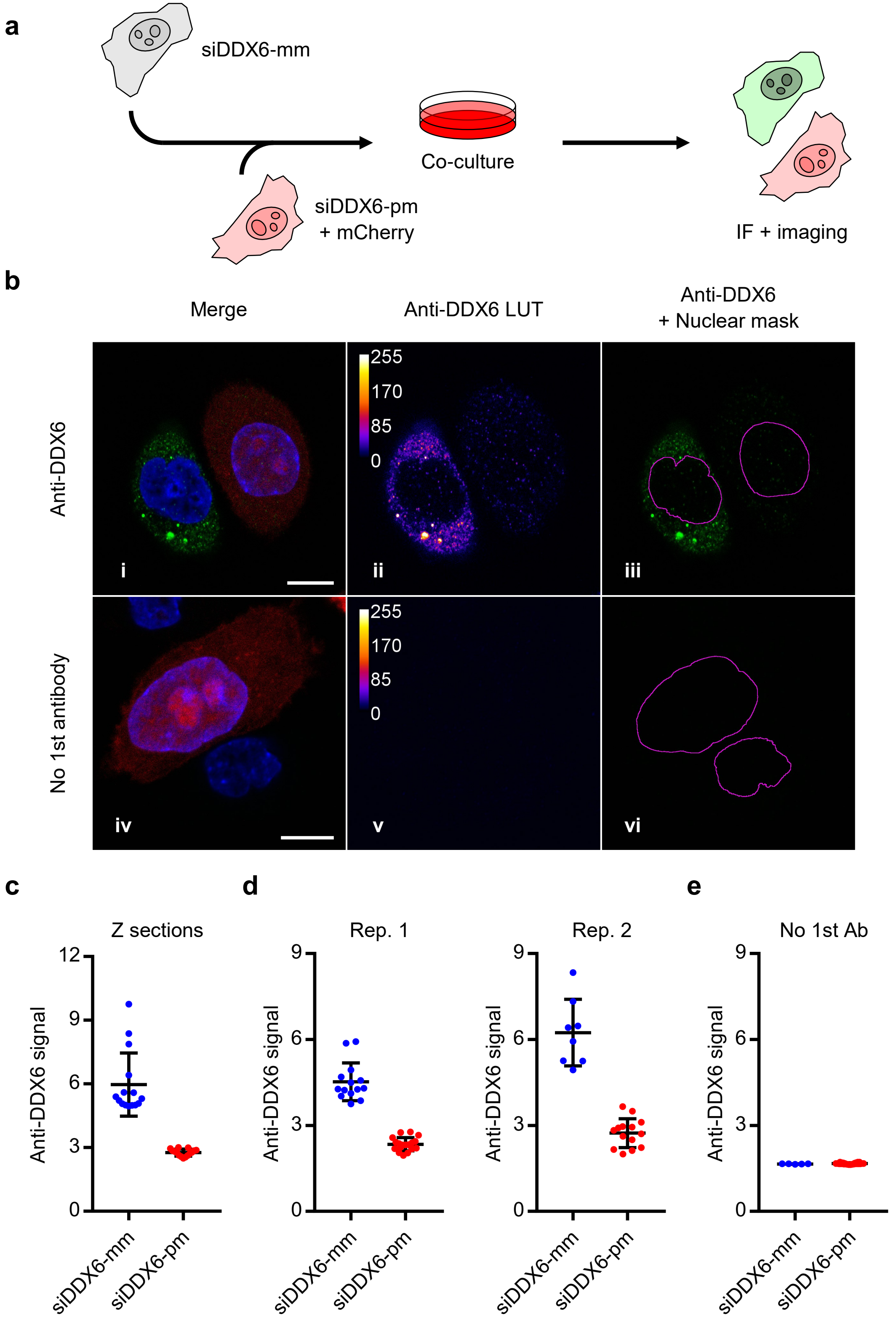
a
siDDX6-mm
siDDX6-pm
+ mCherry
Co-culture
IF + imaging
b
Merge
Anti-DDX6 LUT
Anti-DDX6
+ Nuclear mask
Anti-DDX6
No 1st antibody
255
170
85
0
i
ii
iii
iv
v
vi
c
Z sections
Anti-DDX6 signal
12
9
6
3
0
siDDX6-mm
siDDX6-pm
d
Rep. 1
Rep. 2
e
No 1st Ab

Fig. S4

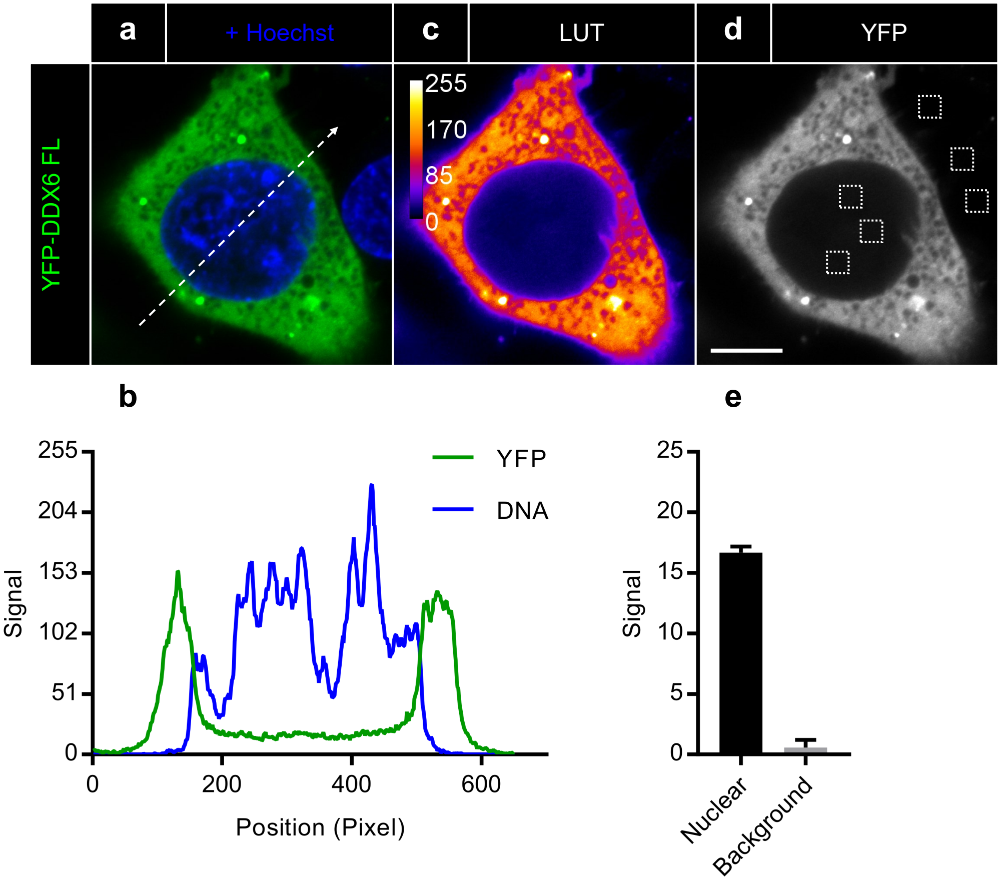
a
+ Hoechst
c
LUT
d
YFP
YFP-DDX6 FL
255
170
85
0
b
Signal
255
204
153
102
51
0
0
200
400
600
Position (Pixel)
YFP
DNA
e
Signal
25
20
15
10
5
0
Nuclear
Background

Fig. S5

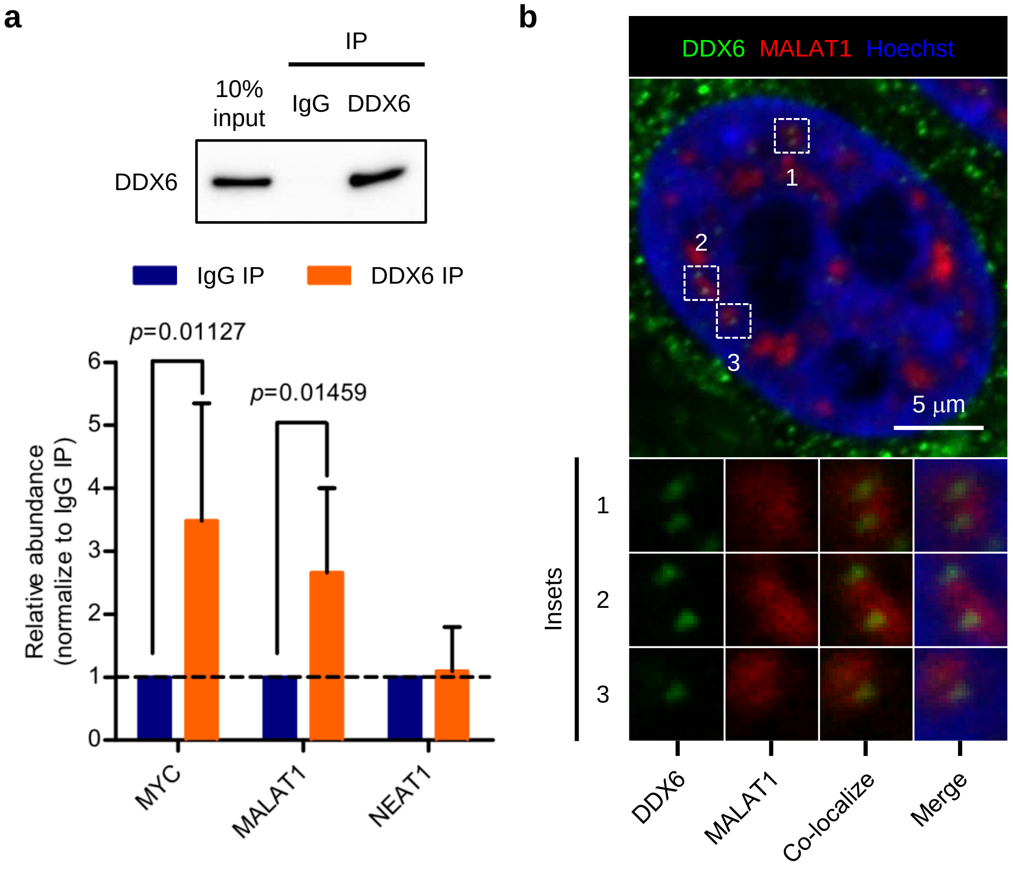
a
IP
10% input
IgG
DDX6
DDX6
IgG IP
DDX6 IP
p=0.01127
p=0.01459
Relative abundance (normalize to IgG IP)
0
1
2
3
4
5
6
MYC
MALAT1
NEAT1
b
DDX6 MALAT1 Hoechst
1
2
3
5 µm
Insets
1
2
3
DDX6
MALAT1
Co-localize
Merge

Fig. S6

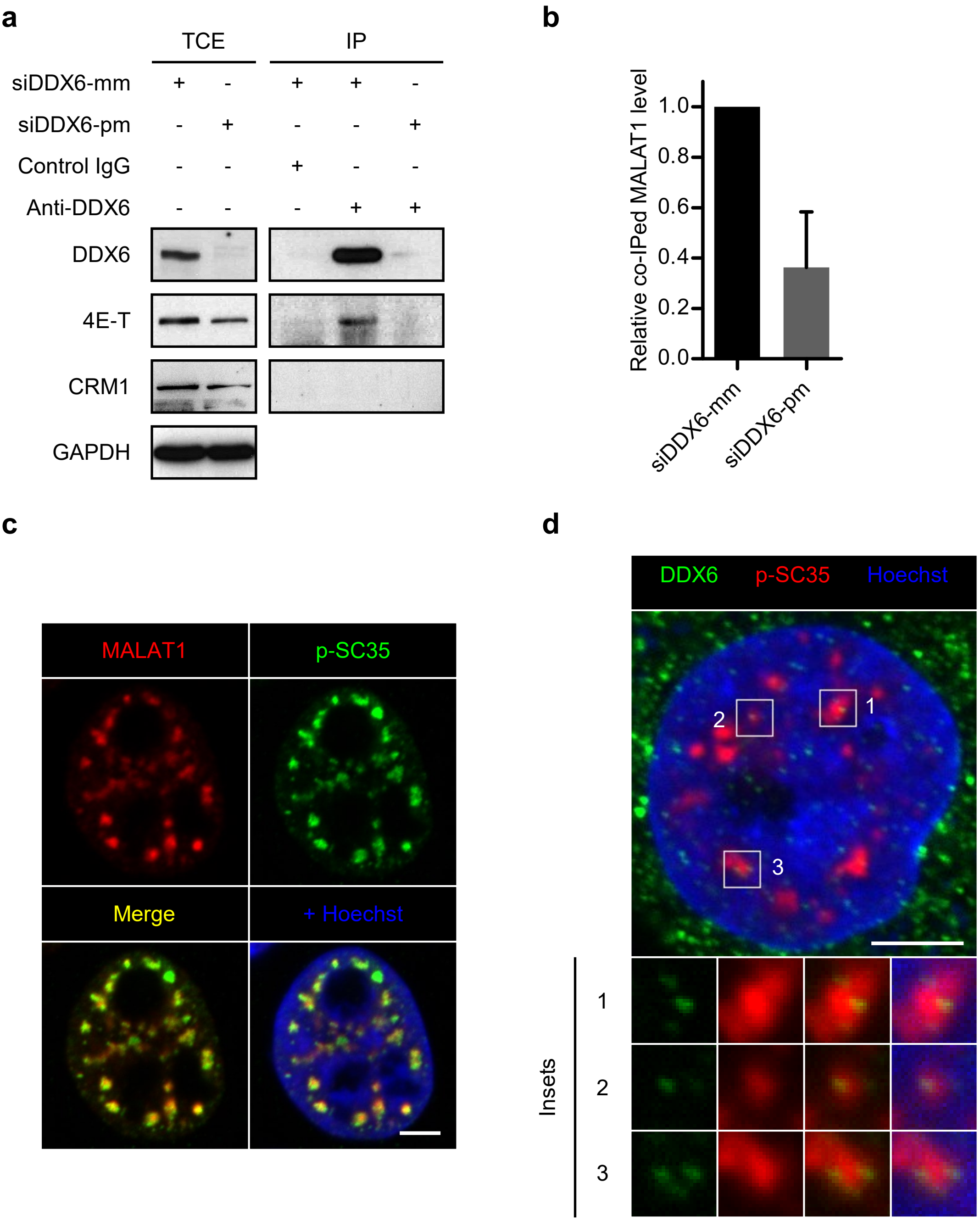
a
TCE
IP
siDDX6-mm
siDDX6-pm
Control IgG
Anti-DDX6
DDX6
4E-T
CRM1
GAPDH
b
Relative co-IPed MALAT1 level
1.0
0.8
0.6
0.4
0.2
0.0
siDDX6-mm
siDDX6-pm
c
MALAT1
p-SC35
Merge
+ Hoechst
d
DDX6
p-SC35
Hoechst
1
2
3
Insets

Fig. S7

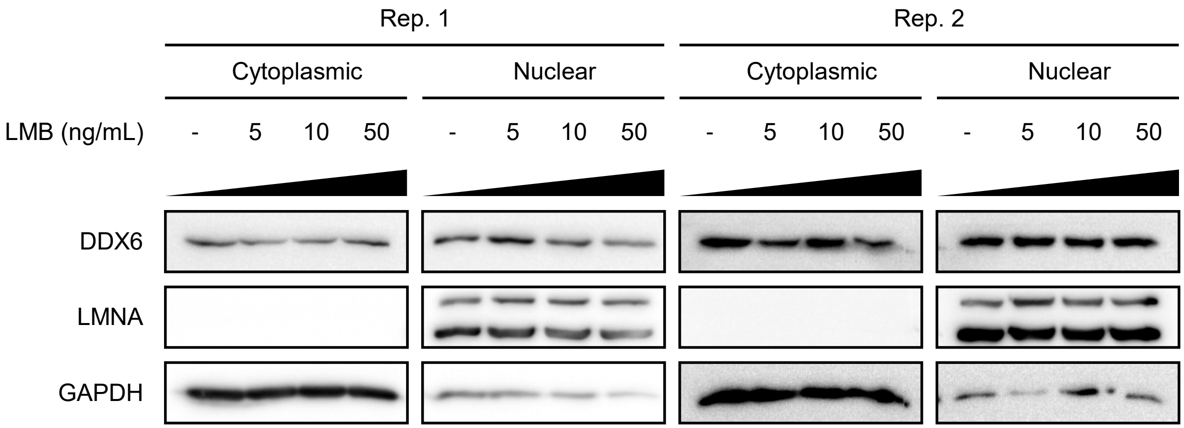

Rep. 1
Rep. 2
Cytoplasmic
Nuclear
Cytoplasmic
Nuclear
LMB (ng/mL)
- 5 10 50
- 5 10 50
- 5 10 50
- 5 10 50
DDX6
LMNA
GAPDH

Fig. S8

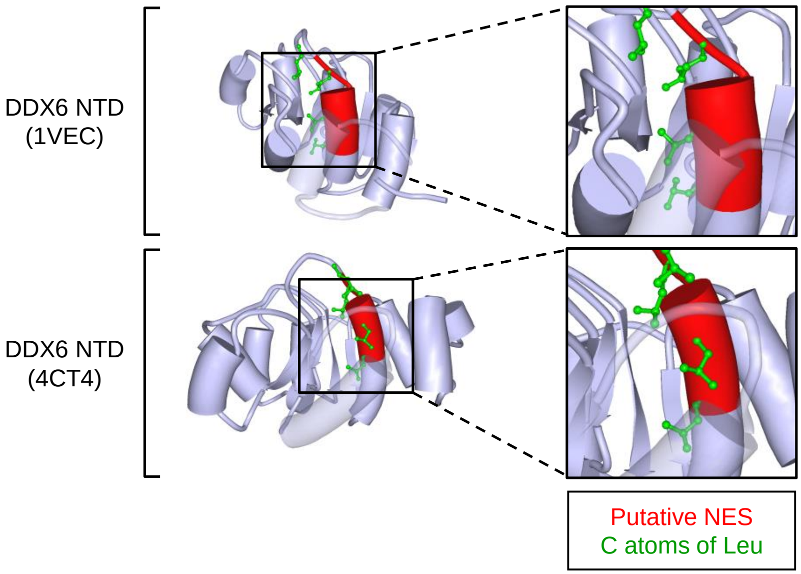

Fig. S9

a

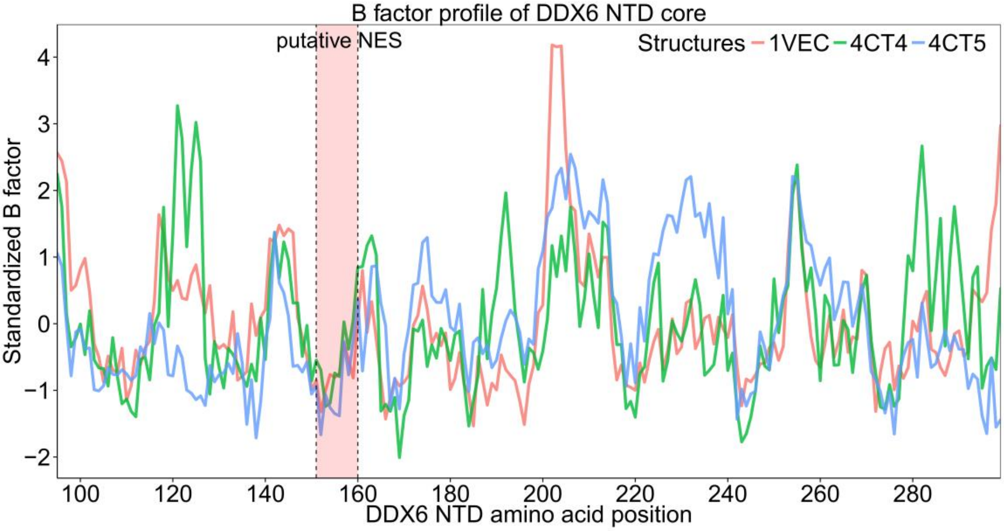

b

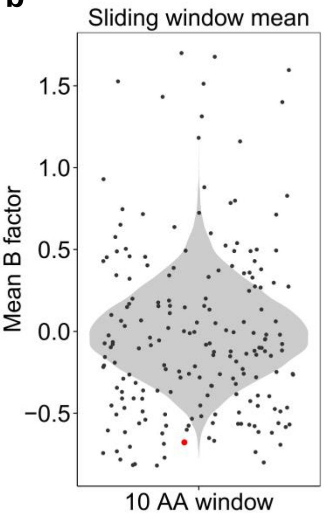

c

```
DDX2A    87 AISILQQIEL  96
DDX2B    88 AISILQQLEI  97
DDX48    93 SISVLQCLDI 102
DDX19A 148 VLAMLSRVEP 157
DDX19B 149 VLAMLSQVEP 158
DDX25  154 VLAMLSRVNA 163
DDX6   151 LIPLLERLDL 160
DDX20  117 STIALDSLVL 126
DDX39A  99 VLATLQQIEP 108
DDX39B 100 VLATLQQLEP 109
```

d

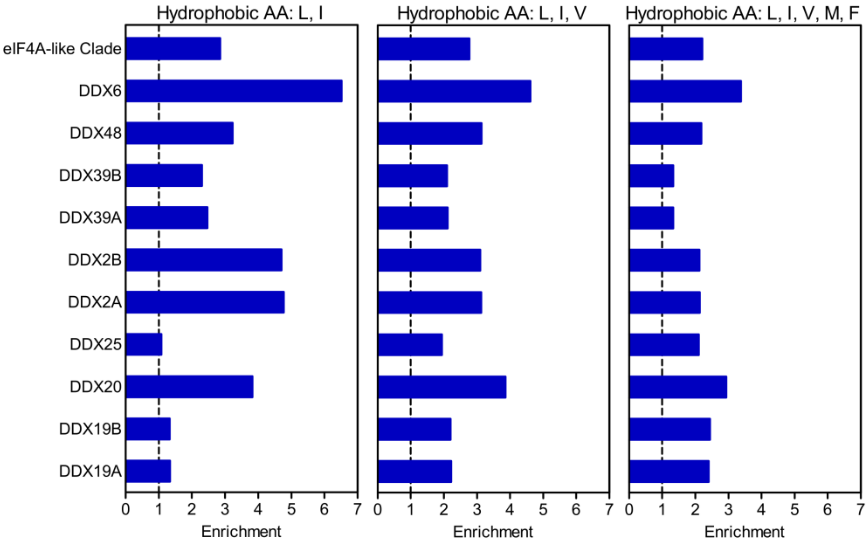

e

eIF4A-like clade

	NES	FL
L, I	36	795
Other AA	64	4031

Odds Ratio 2.851072
P value 3.532e-06

DDX6

	NES	FL
L, I	6	90
Other AA	4	393

Odds Ratio 6.515117
P value 5.063e-03

DDX2A

	NES	FL
L, I	5	70
Other AA	5	336

Odds Ratio 4.77411
P value 2.006e-02

Fig. S10

a

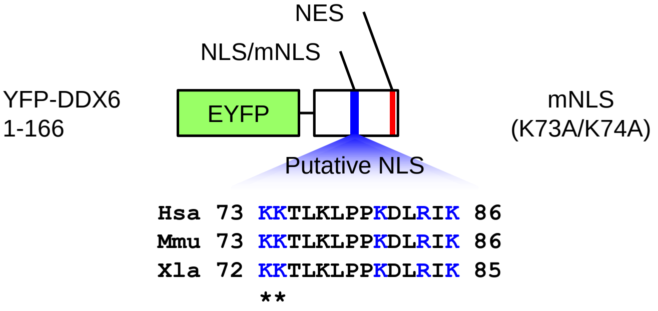

b

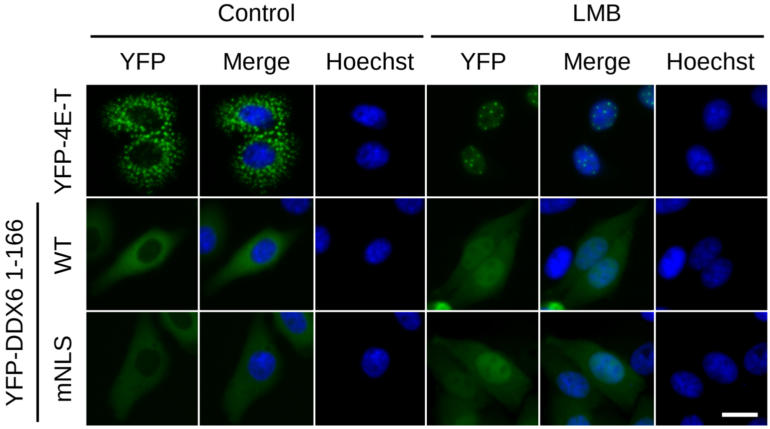

c

DDX6 FL sequence

```
1    MSTARTENPVIMGLSSQNGQLRGPVKPTGGPGGGTQTQQQMNQLKNTNT 50
51   INNGTQQQAQSMTTTIKPGDDWKKTLKLPPKDLRIKTSDVTSTKGNEFED 100
101  YCLKRELLMGIFEMGWEKPSPIQEESIPIALSGRDILARAKNGTGKSGAY 150
151  LIPLLERLDLKKDNIQAMVIVPTRELALQVSQICIQVSKHMGGAKVMATT 200
201  GGTNLRDDIMRLDDTVHVVIATPGRILDLIKKGVAKVDHVQMIVLDEADK 250
251  LLSQDFVQIMEDIILTLPKNRQILLYSATFPLSVQKFMNSHLQKPYEINL 300
301  MEELTLKGVTQYYAYVTERQKVHCLNTLFSRLQINQSIIFCNSSQRVELL 350
351  AKKISQLGYSCFYIHAKMRQEHRNRVFHDFRNGLCRNLVCTDLFTRGIDI 400
401  QAVNVVINFDFPKLAETYLHRIGRSGRFGHLGLAINLITYDDRFNLKSIE 450
451  EQLGTEIKPIPSNIDKSLYVAEYHSEPVEDEKP                 483
```

DBR1 CTD sequence

```
1    DKGQTARATKFLALDKCLPHRDFLQILEIEHDPSAPDYLEYDIEWLTILR 50
51   ATDDLINVTGRLWNMPENNGLHARWDYSATEEGMKEVLEKLNHDLKVPCN 100
101  FSVTAACYDPSKPQTQMQLIHRINPQTTEFCAQLGIIDINVRLQKSKEEH 150
151  HVCGEYEEQDDVESNDSGEDQSEYNTDTSALSSINPDEIMLDEEEDEDSI 200
201  VSAHSGMNTPSVEPSDQASEFSASFSDVRILPGSMIVSSDDTVDSTIDRE 250
251  GKPGGTVESGNGEDLTKVPLKRLSDEHEPEQRKKIKRRNQAIYAAVDDDD 300
301  DDAA                                               304
```

NucPred score

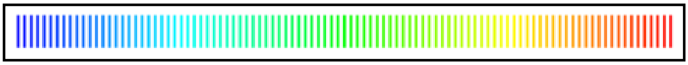

Nuc. - Nuc. +

Fig. S11

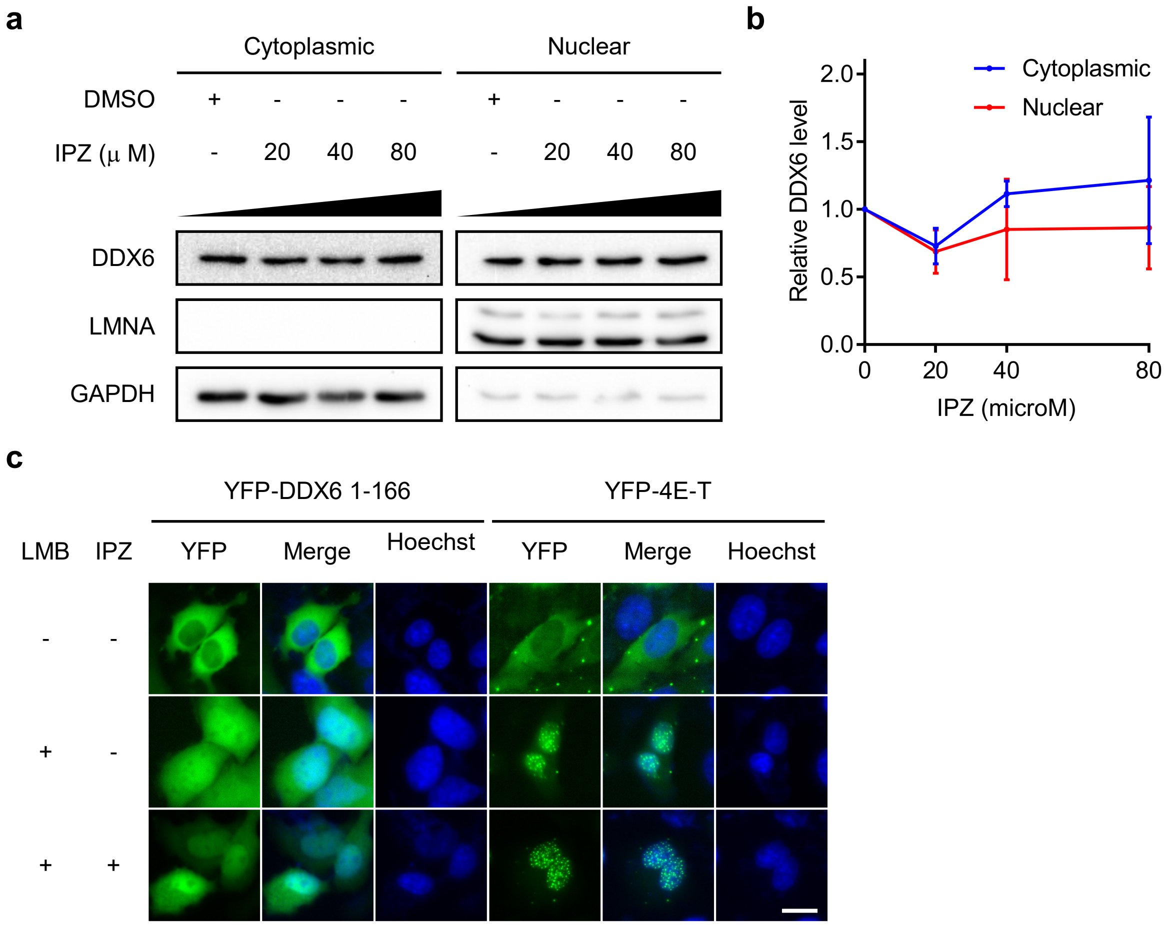
a
Cytoplasmic
Nuclear
DMSO
+
-
-
-
+
-
-
-
IPZ (μ M)
-
20
40
80
-
20
40
80
DDX6
LMNA
GAPDH
b
Cytoplasmic
Nuclear
Relative DDX6 level
2.0
1.5
1.0
0.5
0.0
0
20
40
80
IPZ (microM)
c
YFP-DDX6 1-166
YFP-4E-T
LMB
IPZ
YFP
Merge
Hoechst
YFP
Merge
Hoechst
-
-
+
-
+
+

Fig. S12

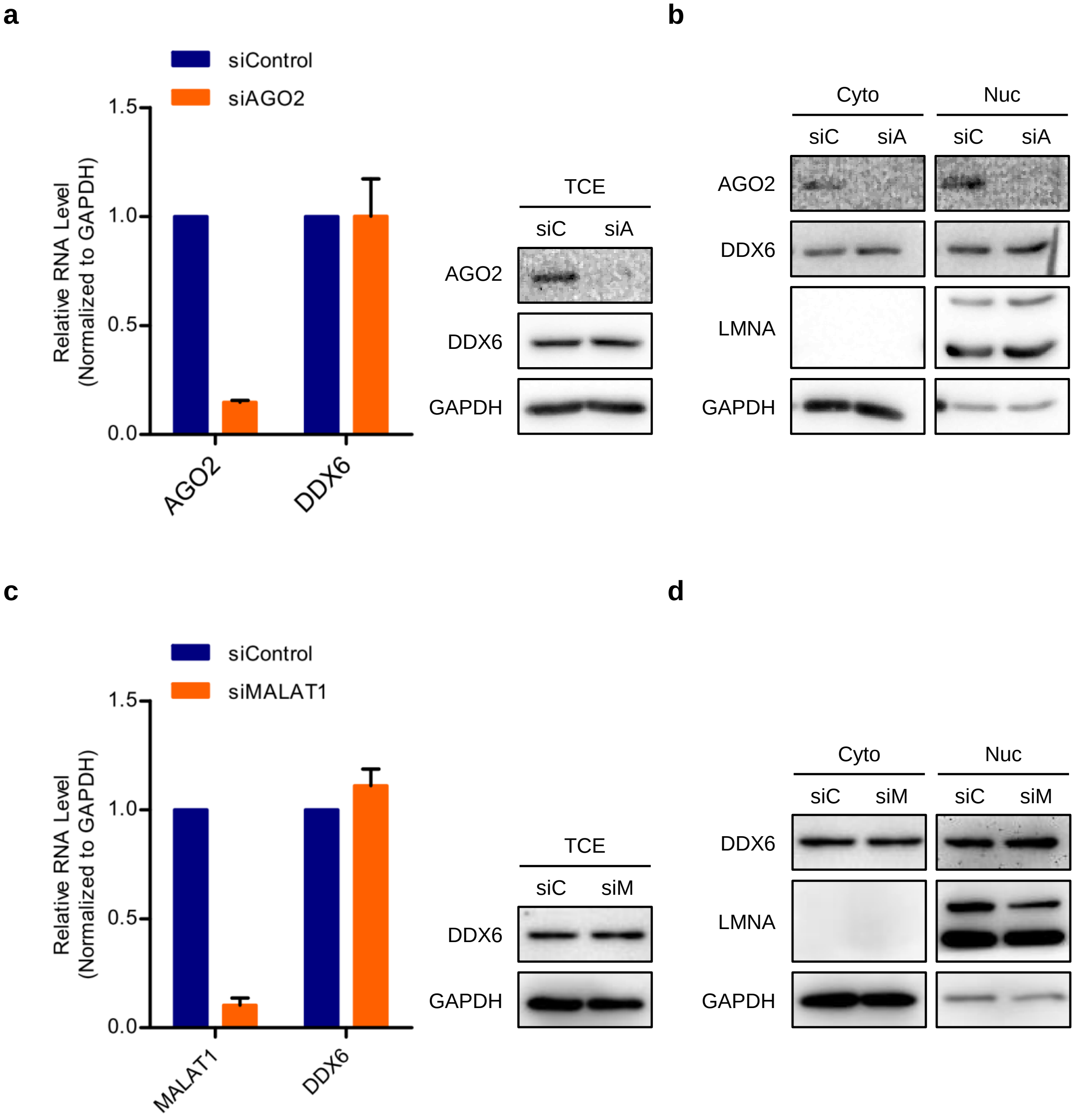
a
siControl
siAGO2
Relative RNA Level
(Normalized to GAPDH)
1.5
1.0
0.5
0.0
AGO2
DDX6
TCE
siC
siA
AGO2
DDX6
GAPDH
b
Cyto
Nuc
siC
siA
siC
siA
AGO2
DDX6
LMNA
GAPDH
c
siControl
siMALAT1
Relative RNA Level
(Normalized to GAPDH)
1.5
1.0
0.5
0.0
MALAT1
DDX6
TCE
siC
siM
DDX6
GAPDH
d
Cyto
Nuc
siC
siM
siC
siM
DDX6
LMNA
GAPDH

Fig. S13

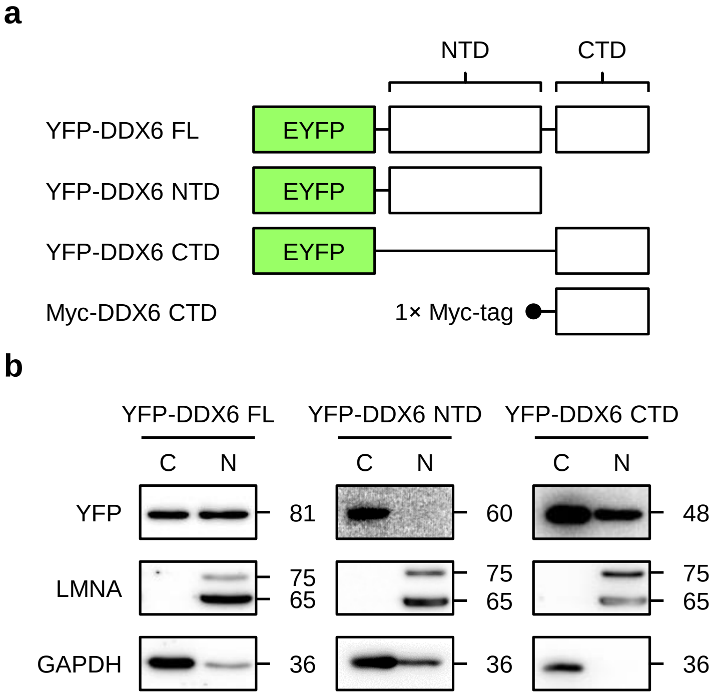
a
NTD
CTD
YFP-DDX6 FL
EYFP
YFP-DDX6 NTD
EYFP
YFP-DDX6 CTD
EYFP
Myc-DDX6 CTD
1× Myc-tag
b
YFP-DDX6 FL
YFP-DDX6 NTD
YFP-DDX6 CTD
C
N
YFP
81
60
48
LMNA
75
65
GAPDH
36
c
YFP
Merge
Hoechst
YFP-DDX6 CTD
Myc IF
Myc-DDX6 CTD
d
YFP-DDX6 CTD
Live cell
4% PFA

Fig. S14

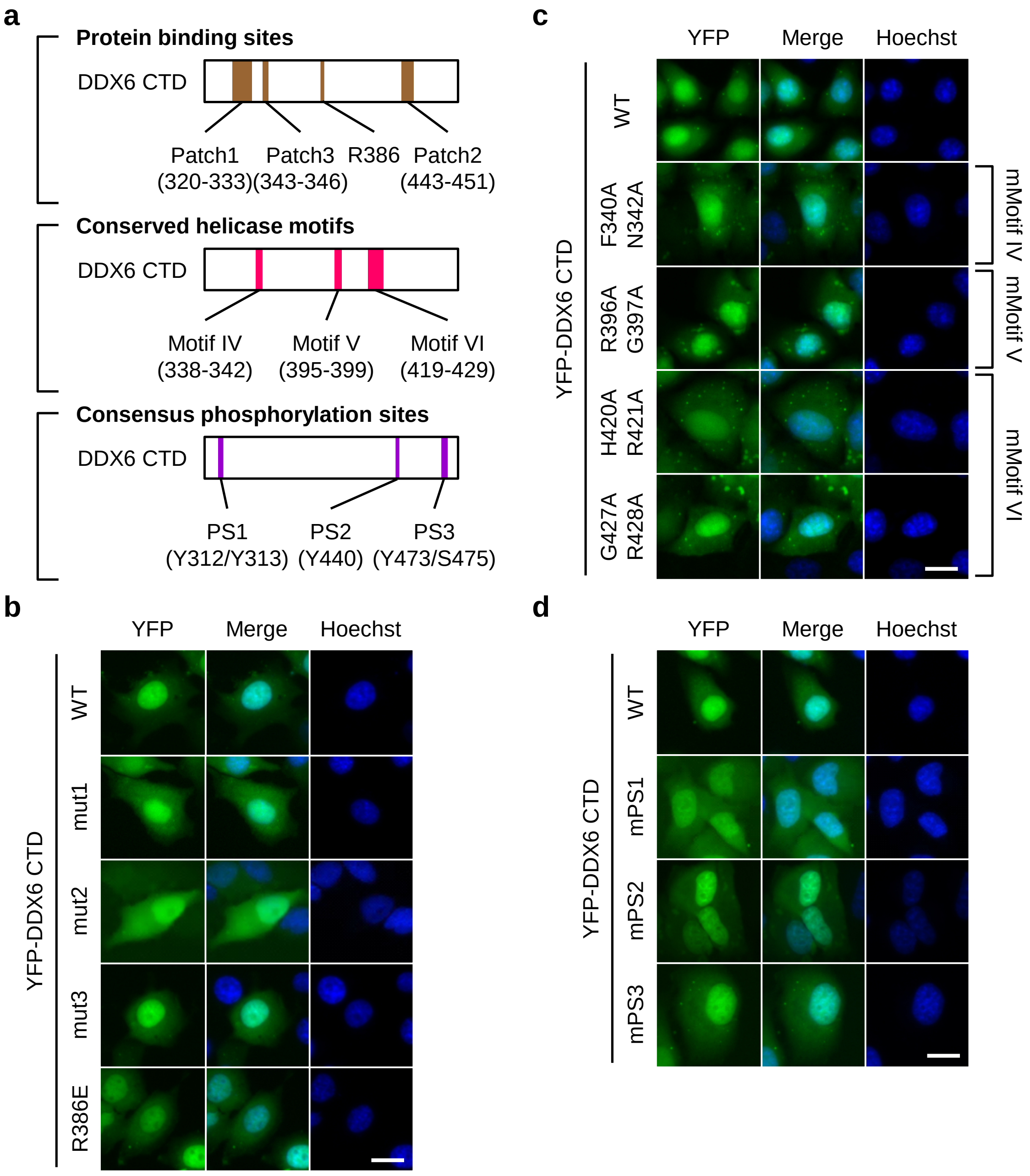

a
Protein binding sites
DDX6 CTD
Patch1
(320-333)
Patch3
(343-346)
R386
Patch2
(443-451)
Conserved helicase motifs
DDX6 CTD
Motif IV
(338-342)
Motif V
(395-399)
Motif VI
(419-429)
Consensus phosphorylation sites
DDX6 CTD
PS1
(Y312/Y313)
PS2
(Y440)
PS3
(Y473/S475)
b
YFP
Merge
Hoechst
YFP-DDX6 CTD
WT
mut1
mut2
mut3
R386E
c
YFP
Merge
Hoechst
YFP-DDX6 CTD
WT
F340A N342A
R396A G397A
H420A R421A
G427A R428A
mMotif IV
mMotif V
mMotif VI
d
YFP
Merge
Hoechst
YFP-DDX6 CTD
WT
mPS1
mPS2
mPS3

Fig. S15

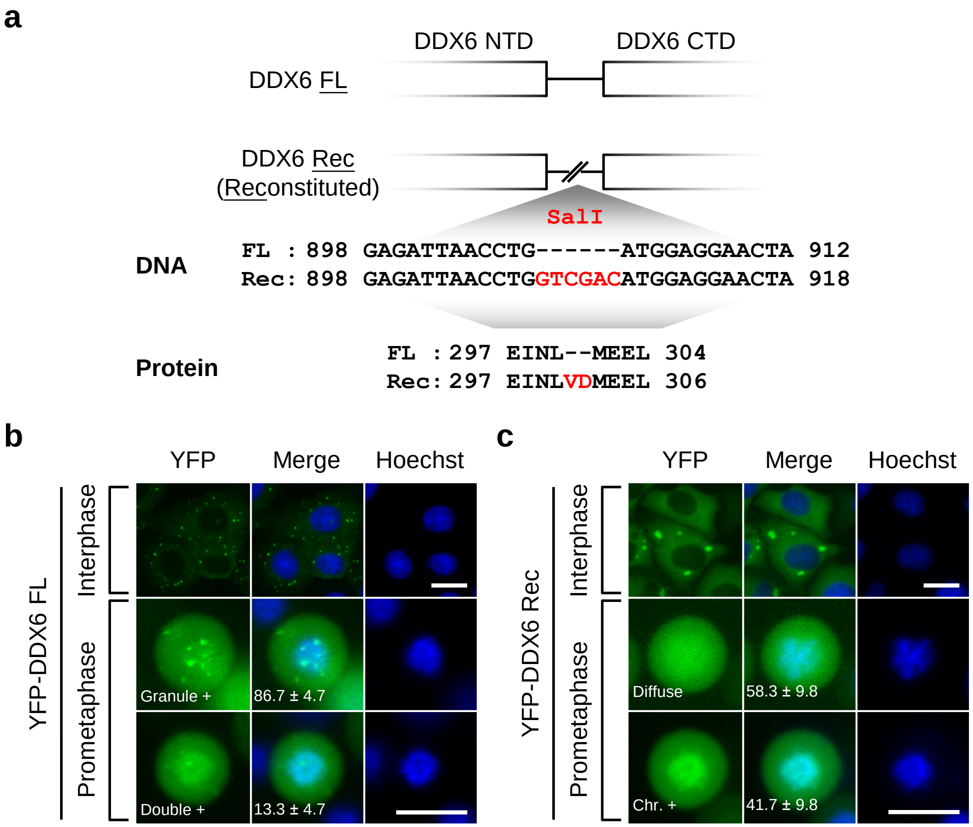

a
DDX6 NTD
DDX6 CTD
DDX6 FL
DDX6 Rec
(Reconstituted)
SalI
DNA
FL : 898 GAGATTAACCTG------ATGGAGGAACTA 912
Rec: 898 GAGATTAACCTGGTCGACATGGAGGAACTA 918
Protein
FL : 297 EINL--MEEL 304
Rec: 297 EINLVDMEEL 306
b
YFP
Merge
Hoechst
YFP-DDX6 FL
Interphase
Prometaphase
Granule +
86.7 ± 4.7
Double +
13.3 ± 4.7
c
YFP
Merge
Hoechst
YFP-DDX6 Rec
Interphase
Prometaphase
Diffuse
58.3 ± 9.8
Chr. +
41.7 ± 9.8

Fig. S16

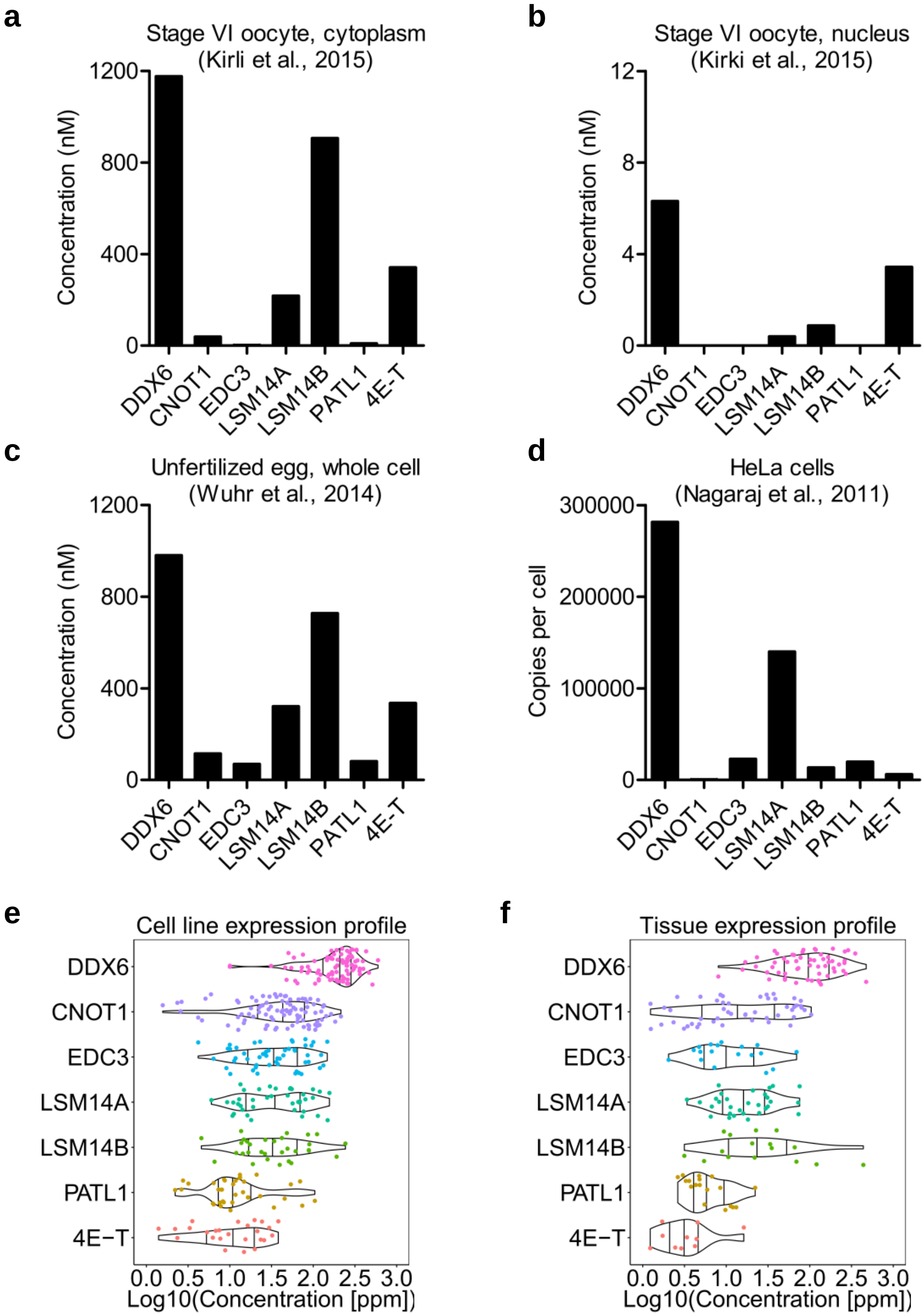
a
Stage VI oocyte, cytoplasm
(Kirli et al., 2015)
Concentration (nM)
0
400
800
1200
DDX6
CNOT1
EDC3
LSM14A
LSM14B
PATL1
4E-T
b
Stage VI oocyte, nucleus
(Kirki et al., 2015)
Concentration (nM)
0
4
8
12
DDX6
CNOT1
EDC3
LSM14A
LSM14B
PATL1
4E-T
c
Unfertilized egg, whole cell
(Wuhr et al., 2014)
Concentration (nM)
0
400
800
1200
DDX6
CNOT1
EDC3
LSM14A
LSM14B
PATL1
4E-T
d
HeLa cells
(Nagaraj et al., 2011)
Copies per cell
0
100000
200000
300000
DDX6
CNOT1
EDC3
LSM14A
LSM14B
PATL1
4E-T
e
Cell line expression profile
DDX6
CNOT1
EDC3
LSM14A
LSM14B
PATL1
4E−T
0.0 0.5 1.0 1.5 2.0 2.5 3.0
Log10(Concentration [ppm])
f
Tissue expression profile
DDX6
CNOT1
EDC3
LSM14A
LSM14B
PATL1
4E−T
0.0 0.5 1.0 1.5 2.0 2.5 3.0
Log10(Concentration [ppm])

Fig. S17

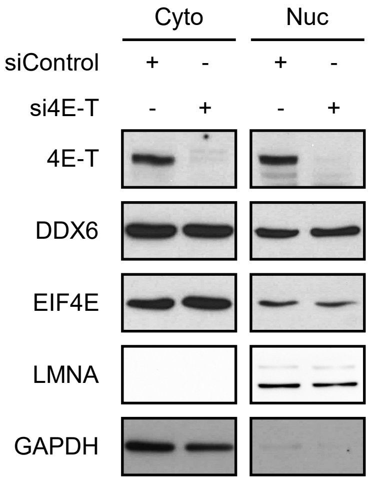

Fig. S18

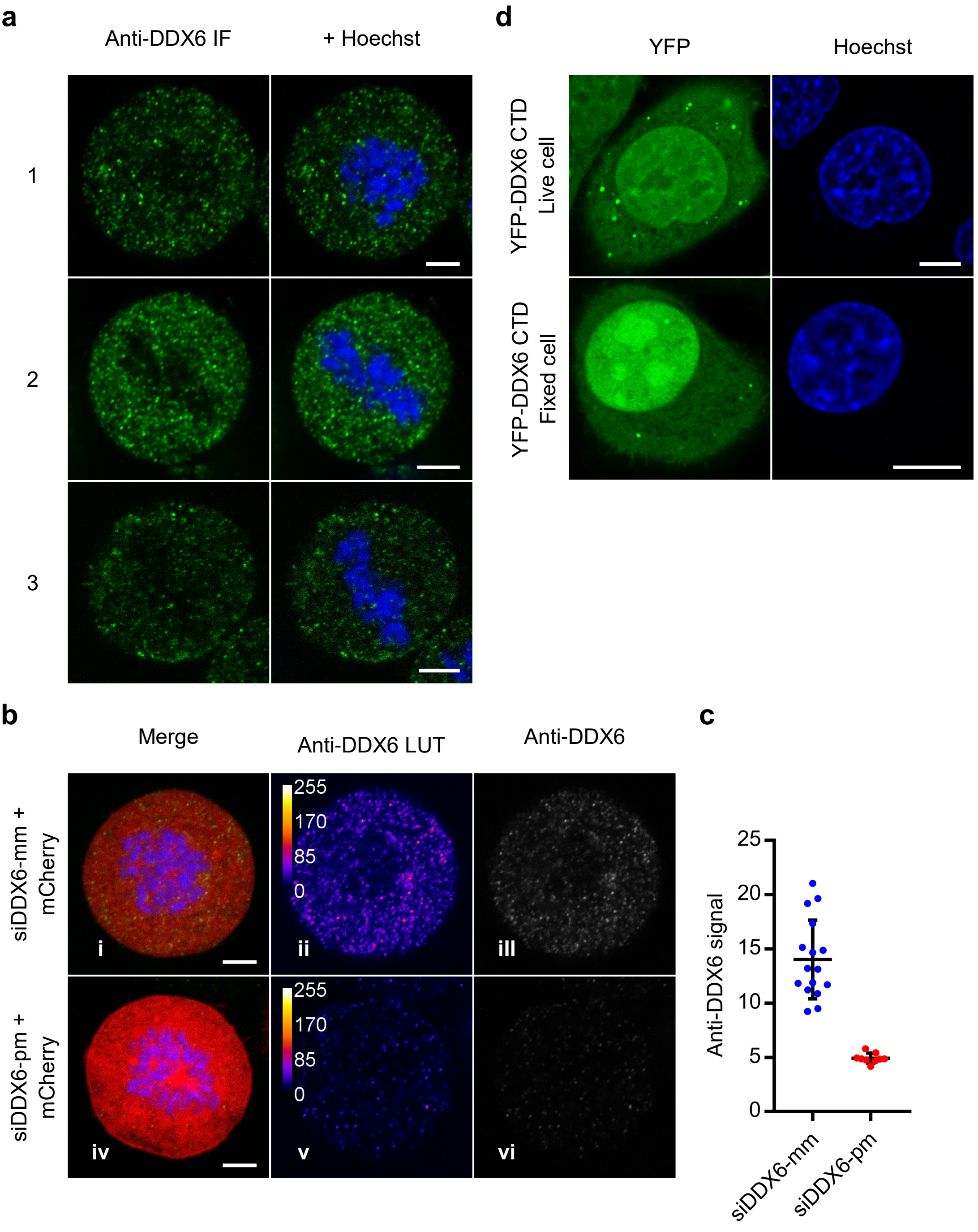
a
Anti-DDX6 IF
+ Hoechst
1
2
3
d
YFP
Hoechst
YFP-DDX6 CTD
Live cell
YFP-DDX6 CTD
Fixed cell
b
Merge
Anti-DDX6 LUT
Anti-DDX6
siDDX6-mm +
mCherry
siDDX6-pm +
mCherry
i
ii
iii
iv
v
vi
255
170
85
0
c
Anti-DDX6 signal
25
20
15
10
5
0
siDDX6-mm
siDDX6-pm

Fig. S19

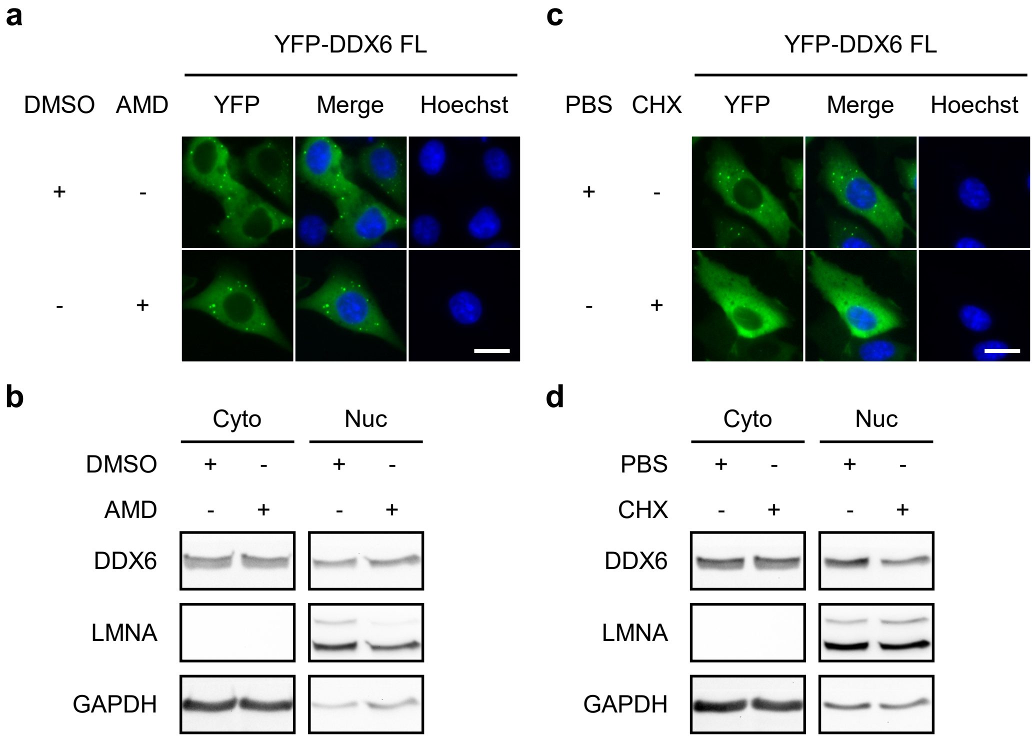
a
YFP-DDX6 FL
DMSO
AMD
YFP
Merge
Hoechst
+
-
-
+
c
YFP-DDX6 FL
PBS
CHX
YFP
Merge
Hoechst
+
-
-
+
b
Cyto
Nuc
DMSO
AMD
DDX6
LMNA
GAPDH
d
Cyto
Nuc
PBS
CHX
DDX6
LMNA
GAPDH

Fig. S20

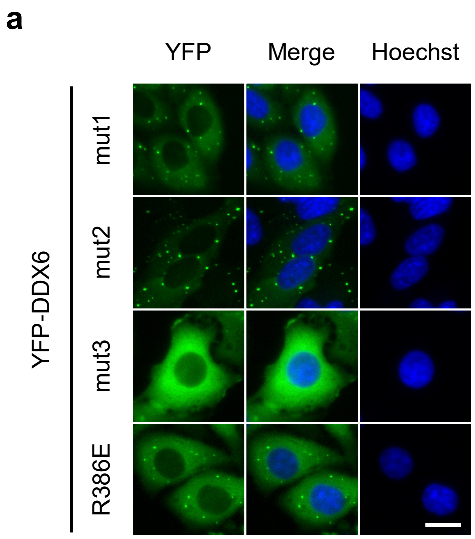
a
YFP
Merge
Hoechst
YFP-DDX6
mut1
mut2
mut3
R386E

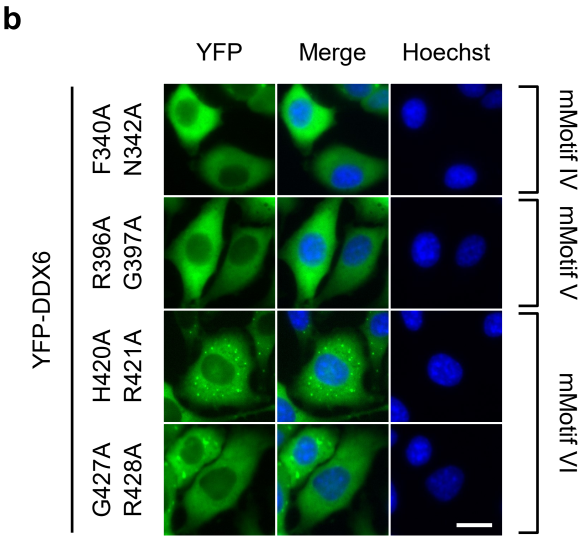
b
YFP
Merge
Hoechst
YFP-DDX6
F340A N342A
R396A G397A
H420A R421A
G427A R428A
mMotif IV
mMotif V
mMotif VI

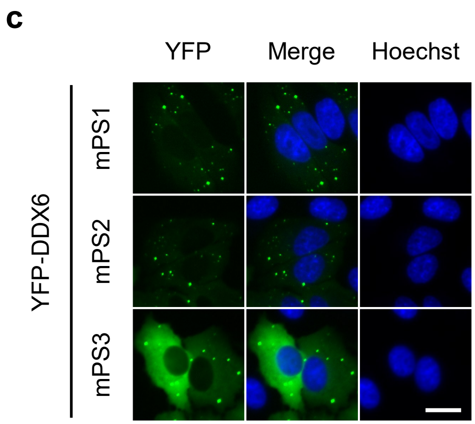
c
YFP
Merge
Hoechst
YFP-DDX6
mPS1
mPS2
mPS3
